# Derived woodiness and annual habit evolved in African umbellifers as alternative solutions for coping with drought

**DOI:** 10.1186/s12870-021-03151-x

**Published:** 2021-08-20

**Authors:** Kamil E. Frankiewicz, Łukasz Banasiak, Alexei A. Oskolski, Anthony R. Magee, Mohammad Alsarraf, Paulina Trzeciak, Krzysztof Spalik

**Affiliations:** 1 Institute of Evolutionary Biology, Faculty of Biology, University of Warsaw, Biological and Chemical Research Centre, Żwirki i Wigury 101, 02-089 Warsaw, Poland; 2 Department of Botany and Plant Biotechnology, University of Johannesburg, PO Box 524, Auckland Park, Johannesburg, 2006 South Africa; 3 Komarov Botanical Institute, Prof. Popov 2, 197376 St. Petersburg, Russia; 4 Compton Herbarium, South African National Biodiversity Institute, Kirstenbosch Research Centre, Rhodes Drive, Cape Town, 7700 South Africa; 5 Faculty of Biology and Veterinary Sciences, Nicolaus Copernicus University, Lwowska 1, 87-100 Toruń, Poland

**Keywords:** Apioideae, Derived woodiness, Habitat, *Nanobubon*, *Notobubon*, Secondary woodiness, Wood anatomy

## Abstract

**Background:**

One of the major trends in angiosperm evolution was the shift from woody to herbaceous habit. However, reversals known as derived woodiness have also been reported in numerous, distantly related clades. Among theories evoked to explain the factors promoting the evolution of derived woodiness are moderate climate theory and cavitation theory. The first assumes that woody habit evolves in response to mild climate allowing for prolonged life span, which in turn leads to bigger and woodier bodies. The second sees woodiness as a result of natural selection for higher cavitation resistance in seasonally dry environments. Here, we compare climatic niches of woody and herbaceous, mostly southern African, umbellifers from the *Lefebvrea* clade to assess whether woody taxa in fact occur in markedly drier habitats. We also calibrate their phylogeny to estimate when derived woodiness evolved. Finally, we describe the wood anatomy of selected woody and herbaceous taxa to see if life forms are linked to any particular wood traits.

**Results:**

The evolution of derived woodiness in chamaephytes and phanerophytes as well as the shifts to short-lived annual therophytes in the *Lefebvrea* clade took place at roughly the same time: in the Late Miocene during a trend of global climate aridification. Climatic niches of woody and herbaceous genera from the Cape Floristic Region overlap. There are only two genera with distinctly different climatic preferences: they are herbaceous and occur outside of the Cape Floristic Region. Therefore, studied herbs have an overall climatic niche wider than their woody cousins. Woody and herbaceous species do not differ in qualitative wood anatomy, which is more affected by stem architecture and, probably, reproductive strategy than by habit.

**Conclusions:**

Palaeodrought was likely a stimulus for the evolution of derived woodiness in the *Lefebvrea* clade, supporting the cavitation theory. The concurrent evolution of short-lived annuals withering before summer exemplifies an alternative solution to the same problem of drought-induced cavitation. Changes of the life form were most likely neither spurred nor precluded by any qualitative wood traits, which in turn are more affected by internode length and probably also reproductive strategy.

**Supplementary Information:**

The online version contains supplementary material available at 10.1186/s12870-021-03151-x.

## Background

Angiosperms – unlike their closest extant relatives – are very diverse in terms of life span (annuals to perennials), reproductive strategy (monocarpic and polycarpic), and the degree of woodiness (from species completely lacking secondary growth to enormous trees with secondary xylem meters thick). The habit of their most recent common ancestor remains obscure, but most likely it possessed a functional cambium producing a limited amount of wood [1, 2]. This interpretation is in agreement with the *dark and disturbed* hypothesis assuming that the first angiosperms occupied constantly changing understorey of the (sub) tropical forests [3]. In such an environment, a shorter life span and smaller, quickly-developing body are advantageous as they allow to complete life cycle in narrow intervals between sequent disturbances threatening plant survival. The shortening of life span allows for deposition of only a small amount of wood, but the opposite is not mandatory: e.g., *Salix arctica* easily outlives arborescent species, while producing only a fraction of wood they do [4]. Indeed, the evolution from more woody ancestors to less woody descendants has probably been the major trend in the angiosperm evolution, resulting in numerous herbaceous lineages able to occupy unstable niches hardly available for their woodier relatives [5]. The evolution towards herbaceousness, however, has not been the sole direction. A constant increase in the deposition of xylem resulted in ancestrally (primarily) woody groups (e.g., Lauraceae), while other taxa – after a period of herbaceousness – reverted the trend and gave rise to woody descendants: a phenomenon known as (phylogenetically) derived or secondary woodiness [6]. Derived woodiness evolved in multiple, distantly related lineages [6, 7], evidencing that the ability to produce a significant amount of wood is retained throughout the ‘herbaceous phase’ in evolution. In fact, a complete cylinder of secondary xylem develops in most non-monocot herbs making the distinction between herbaceous and woody taxa fuzzy [8, 9].

What prohibits non-monocot herbs from a complete loss of functional cambium? First, it is possible that genes regulating cambial activity show pleiotropic effects and their complete loss would upset other plant functions [10]. Second, minimal cambial activity may be indifferent to overall plant fitness and, therefore, may not be counter-selected. Irrespectively of the cause, this retention makes angiosperms highly adaptable, allowing them to shift the amount of produced wood in response to the current evolutionary pressures and has been tapped as a likely key to their success [2, 11]. Simultaneously, it is worth noting that even a loss of genes preferentially expressed during xylogenesis followed by a complete loss of cambial activity does not necessarily preclude the evolution of a range of life forms – from annual herbs to trees – as is evidenced in monocots [12–14].

As mentioned above, a longer life span allows for (but is not synonymous with) the production of a prominent amount of wood. In fact, experimental studies with *A. thaliana* showed that the prolongation of vegetative growth by manual clipping of flowers or by knocking-out genes responsible for the initiation of flowering leads to an increased deposition of secondary xylem [15, 16]. The prolongation of life span is more likely in stable, moderate environments, where a plant has higher chances of completing its life cycle without encountering seasonal droughts or freezing [11]*.* Such conditions often prevail on (sub) tropical oceanic islands. Therefore, it is reasonable to expect that herbs – after their arrival from the mainland to such an island – will often prolong their life spans and simultaneously increase the amount of wood, resulting in derived woody descendants (in this case named *insular woody*): a notion first proposed by Carlquist and termed *moderate climate theory* [17]. Moreover, the oceanic islands are often devoid of large herbivores, liberating newcomers from yet another detrimental factor observed on the mainland [18]. The conditions similar to insular ones are also found on slopes of high, tropical mountains. These *sky islands* have been shown to boast a diversity of derived woody taxa [10, 19–21], supporting the role of stable, moderate climates in the evolution from herbs to woody life forms.

However, a survey of the Canary Islands flora [7] exposed that most of the derived woody taxa do not occupy moderate niches – jarring with the moderate climate theory – but are rather confined to markedly dry, lowland areas. One explanation for this apparent incongruence is that woodier taxa may be more resistant to drought-induced embolism (also referred to as ‘cavitation’: a formation of air bubbles in wood hindering water transport and potentially leading to lower photosynthetic rates) and therefore also better adapted to dry habitats than their herbaceous relatives [22]. In fact, a comparison of embolism-resistance between insular woody *Argyranthemum* (Asteraceae) and their herbaceous cousins [23, 24], as well as among woodier and less woody herbaceous crucifers [25, 26], showed that species and specimens producing more wood are less prone to developing cavitation. The correlation between a higher amount of wood and drought resistance is not, however, so straightforward. For example, derived woody Rubiaceae occupy markedly variable habitats: from very dry to noticeably wet [27], and some of them simultaneously favour tropical upland areas [20], while virtually all species of Balsaminaceae (herbaceous and derived woody) grow exclusively in wet (to semi-aquatic) conditions [28]. Moreover, woodier habits may not universally convey more embolism-resistance: a broad comparison of herbaceous and woody angiosperms showed that both groups have similar ranges of drought tolerance (this study was, however, biased towards grasses) [29].

From the above, it becomes evident that derived woodiness evolves in response to various – sometimes antithetic – factors. Nevertheless, two main trends emerge: (1) moderate climate (sometimes coupled with the lack of large herbivores) liberates plants from seasonality, allowing them to prolong their vegetative growth and to benefit from reusable bodies [2, 30], and (2) drought-induced embolism may be so detrimental that natural selection favours more resistant and therefore woodier habits. These two explanations have been preferred in recent years [2, 7, 21], but they are not the only ones. Darwin proposed that insular woodiness results from fierce competition for access to light among herbaceous newcomers [31]. Wallace, on the other hand, stressed that longer-lived (and therefore woodier) habits are advantageous in pollinator-scarce environments [32]: perennial taxa have more time to interact with pollinators, thereby avoiding the detrimental consequences of selfing [33, 34].

The cases of derived woodiness have also been found in Apioideae, which is the largest of four subfamilies currently recognised within the family Apiaceae [35] (for alternative circumscription treating members of subfamily Saniculoideae as tribes within Apioideae see [36]). Subfamily Apioideae is predominately a herbaceous group, and its most recent common ancestor was also most likely a herb [37–39]; therefore, all cases of woodiness in this subfamily can be safely recognised as derived. These are, however, not numerous. Markedly woody habit dominates in mainly southern African tribe Heteromorpheae [40], but otherwise it is restricted to only a few species per clade: in the monotypic tribe Marlothielleae from Namibia, predominantly Mediterranean Bupleureae [41], Mesoamerican *Arracacia* clade of tribe Selineae, insular species of carrots (*Daucus*, subtribe Daucinae) from Macaronesia [42], mostly circum-Mediterranean and northern African relatives of celery (three species of shrubby *Deverra* are also found in southern Africa; Apieae) [43, 44], sub-Saharan *Lefebvrea* clade of Tordylieae [37, 38, 45–47], and also in few isolated lineages of the tribes Selineae and Echinophoreae (*Angelica lignescens*, *Nirarathamnos asarifolius*, *Pycnocycla* spp., *Xyloselinum* spp.) [48, 49].

To date, the evolution of derived woodiness in Apioideae has been studied in the Macaronesian carrots and the Mediterranean and African Apieae [42, 43]. In the first case, it was suggested that derived woodiness evolved in response to moderate, aseasonal climate. In woody Apieae, a selection for higher embolism-resistance seems to be a more likely explanation. These two cases further emphasise how different may be the causes driving the onset of derived woodiness in related clades. In the present study, we used the *Lefebvrea* clade (Tordylieae, Apioideae, Apiaceae) to explicitly test for the link between the evolution of derived woodiness and climatic niche.

Tribe Tordylieae comprises three clades: mostly Eurasian Tordyliinae, Caucasus–Arabian *Cymbocarpum* clade, and sub-Saharan *Lefebvrea* clade [50]. Most species are herbaceous, while woody forms are found in two genera of the *Lefebvrea* clade: *Nanobubon* (suffrutices with woody subterranean stems) and *Notobubon* (shrublets and shrubs, occasionally also medium-size trees) [37, 45, 46]. Using the *Lefebvrea* clade as a model, we checked whether the evolution of woody habit is coupled with a preference for any specific climatic niche (e.g., dry or moderate habitats) or whether the habit evolved independently of the occupied niche. For this, we estimated the time of derived woodiness onset and reconstructed the ancestral habit (life span, reproductive strategy, and life form). We used Raunkiaer’s [51] life forms as a proxy for the degree of woodiness, reasoning that bigger life forms (chamaephytes, phanerophytes) usually require stronger mechanical support, and therefore are also more likely to deposit more wood than their herbaceous – therophytic or hemicryptophytic – relatives. This is the same approach we embraced in our earlier research on derived woodiness in apioids as all the studied species retained cambial activity [42, 43]. We also examined secondary xylem anatomy of woody and herbaceous representatives of Tordylieae (with a major focus on the *Lefebvrea* clade) and woody outgroup (*Pycnocycla*, tribe Echinophoreae) to assess if these two habits differ in qualitative wood traits. Finally, we estimated the climatic niches occupied by the genera of the *Lefebvrea* clade. Within this clade, marked contrasts in life form are at the generic level, while within the genera, life forms are rather homogenous. In this way, we assessed whether woody genera (*Nanobubon*, *Notobubon*) show a preference for drier or more moderate habitats than their herbaceous cousins.

## Results

### Phylogenetic relationships and the age of derived woodiness

**Fig. 1:**
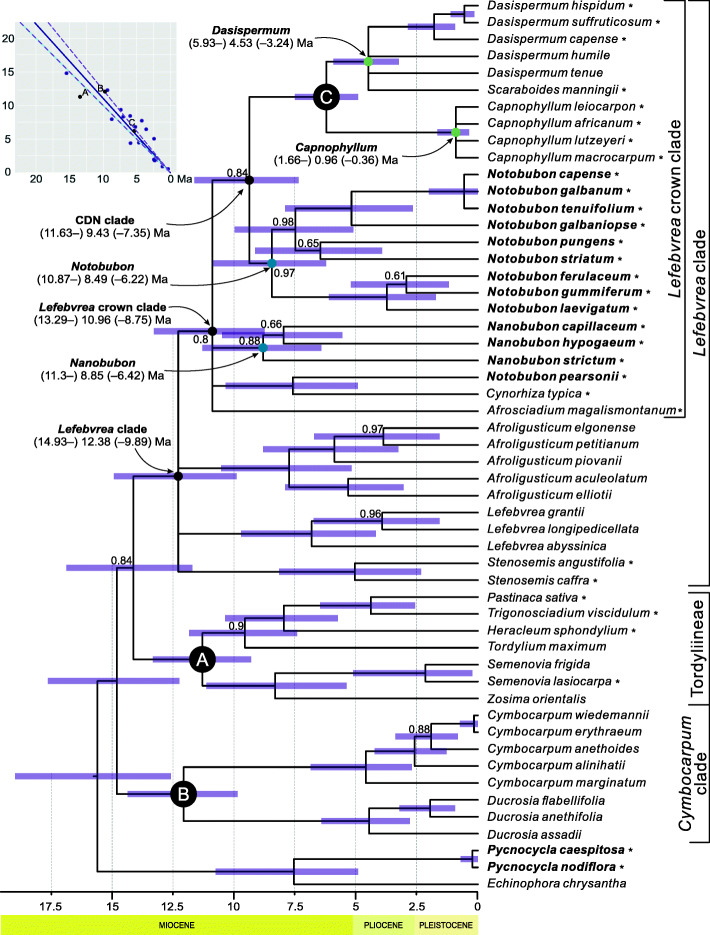
A 50% majority rule consensus tree summarising Bayesian MCMC analyses of Tordylieae and outgroups using MrBayes. Scatter plot represents correlation between median ages of corresponding nodes in primary (abscissa axis) and secondary (ordinate axis) calibrated trees. Solid and dashed violet lines represent major axis with 95% confidence interval compared to the blue line expected under assumption of no systematic bias in age estimations. A, B, and C are calibration points. Violet bars show 95% highest posterior density intervals for age distributions. Crown ages of selected clades are provided and marked with blue (woody), green (herbaceous), or black (woody and herbaceous life forms) dots. For clarity, only posterior probability values lower than 1.0 are shown. Woody (chamaephytic) species are in boldface, species considered for wood anatomy are marked with asterisks. The time scale is in million years ago. Major clades are bracketed. CDN stands for *Capnophyllum*–*Dasispermum*–*Notobubon* clade

The phylogenetic relationships resolved with the maximum likelihood (ML) and Bayesian inference (BI) were very similar and the three main clades of Tordylieae were retrieved (Fig. 1). In the ML analysis, *Lefebvrea* was a sister to the remaining genera of the *Lefebvrea* clade (albeit with poor bootstrap support, BS = 40%; ML phylogenetic tree is available online, see ‘Supplementary information’ under this article). The remaining intergeneric relationships within this clade remained practically unresolved (BS < 50%), except for *Dasispermum* and *Capnophyllum* being sisters (BS = 91%). All genera were monophyletic with two exceptions: monospecific *Scaraboides* was placed within *Dasispermum* (BS = 90%), and *Notobubon pearsonii* did not group with its congeners but was a sister to *Cynorhiza typica* (BS = 51%), the sole representative of *Cynorhiza* in our study.

In the Bayesian 50% majority rule consensus tree (Fig. 1), the intergeneric relationships within the *Lefebvrea* clade were unresolved except for a sister relationship between *Dasispermum* and *Capnophyllum* (PP = 1.0). This clade was in turn a sister to *Notobubon* (PP = 0.84). Similar to the ML tree, *Scaraboides manningii* was resolved as a relative to *Dasispermum*, and *Notobubon pearsonii* was sister to *Cynorhiza typica* (PP = 1.0).

A systematic bias of the secondary divergence time estimates in comparison to primary calibration was negligible as demonstrated by the 95% confidence interval for the slope parameter of the major axis spanning 0.99–1.23 (Fig. 1). The age of Tordylieae was resolved at 14.88 million years (My) with 95% highest posterior density (HPD) interval of 17.64–12.24. The *Lefebvrea* clade originated 12.38 My ago (HPD: 14.93–9.89), and its crown clade (i.e., excluding *Afroligusticum*, *Lefebvrea*, and *Stenosemis*) was resolved as 10.96 My old (HPD: 13.29–8.75). *Nanobubon* diversified (i.e., its crown age) 8.85 (HPD: 11.3–6.42) My ago. The most recent common ancestor of *Capnophyllum*, *Dasispermum*, and *Notobubon* (hereafter termed CDN) lived 9.43 My ago (HPD: 11.63–7.35), and these three genera subsequently diversified (i.e., their crown ages): 0.96 (1.66–0.36), 4.53 (5.93–3.24), and 8.49 (10.87–6.22) My ago, respectively.

### Phylogenetic character mapping

The results of the ancestral states reconstruction need to be interpreted with caution: although the life span, life form, and reproductive strategy were estimated independently, they are biologically interrelated. Annuals can survive an unfavourable period as seeds (therophytes) or seedlings (hemicryptophytes), but they are always monocarpic. Biennials are monocarpic hemicryptophytes. Perennial taxa are mono- or polycarpic, and range from hemicryptophytes, through chamaephytes to phanerophytes, but can never be therophytes. The applicability of life history characters for taxonomic delineation within the *Lefebvrea* clade was considered elsewhere [37, 38].

**Fig. 2:**
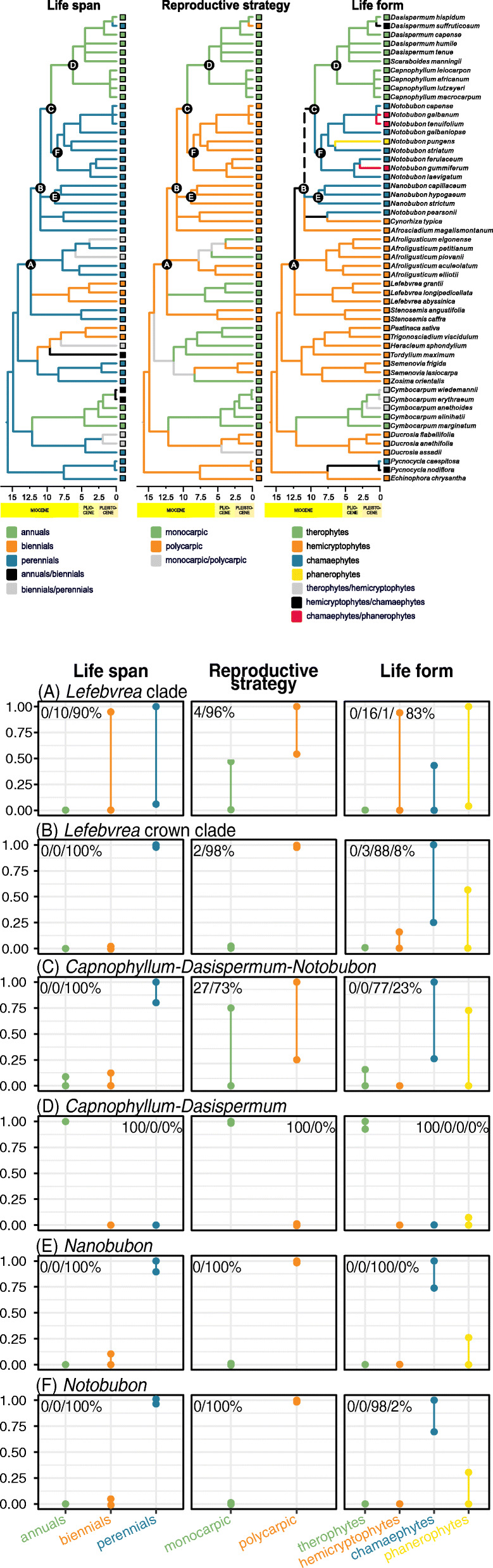
Evolution of life span, reproductive strategy, and life form reconstructed for the entire phylogenetic tree using maximum parsimony (branch colours; the first panel) and for its selected nodes using maximum likelihood (plots and percentage values; the second panel). Letters A–F correspond to the nodes which states were reconstructed with ML and shown in the second panel. The plots show 95% highest posterior density intervals of relative likelihood for each state reconstructed in a given node. The percentage values are numbers of times a given state was reconstructed in a given node out of a sample of 180,000 phylogenetic trees from the Bayesian analysis. Dashed line marks a branch for which MP returned three equally likely states (therophyte, hemicryptophyte, chamaephyte)

The ancestral states reconstructed with maximum parsimony (MP) and ML approaches differed to various degrees as only the ML method considers branch lengths (Fig. 2). Moreover, MP was carried out over a consensus tree while for ML we employed a posterior sample of 180,000 ultrametric phylogenetic trees from the Bayesian analysis. In our summary of ML results, we considered how many times a given state was reconstructed in a given node across a sample of posterior phylogenies (percentage values in the second panel of Fig. 2), as well as the relative support for each state (plots of the 95% highest posterior density intervals in the second panel of Fig. 2); when ML reconstruction differed from MP method, we arbitrarily used MP as a decisive factor.

The most recent common ancestor of the *Lefebvrea* clade (node A in Fig. 2) was reconstructed as a polycarpic, perennial hemicryptophyte. The most recent common ancestor of the crown group of this clade (node B) retained the same reproductive strategy and life span, but its life form can be interpreted in two ways. (1) It evolved a bigger, chamaephytic habit, and such a combination (i.e., polycarpic, perennial chamaephyte) was subsequently ancestral for *Nanobubon* (node E), as well as for CDN (node C in Fig. 2). This combination was also retained in *Notobubon* (node F), while the most recent common ancestor of *Capnophyllum* and *Dasispermum* (node D) underwent a life span shortening combined with the development of smaller body and a reduction in the number of fruitings (i.e., monocarpic, annual therophyte). Alternatively, (2) the most recent common ancestor of the *Lefebvrea* clade crown group (node B) and the most recent common ancestor of CDN (node C) can be interpreted as polycarpic, perennial hemicryptophytes (what is supported by hemicryptophytic habit retained along most of the phylogeny backbone in MP analysis). The descendants of CDN subsequently took two, divergent evolutionary pathways: the ancestor of *Notobubon* (node F) evolved chamaephytic habit, while retaining polycarpic reproductive strategy and perennial life span, and the most recent common ancestor of *Capnophyllum* and *Dasispermum* (node D) underwent a life span shortening and reduction in body size arriving at monocarpic, annual therophyte. In this interpretation, chamaephytes evolved at least twice, independently: in *Nanobubon* and in *Notobubon* (and, if paraphyly of *Notobubon* is confirmed, also in *Notobubon pearsonii*).

### General observations on wood anatomy in Tordylieae and the outgroup

The detailed wood anatomical descriptions of the studied species (Additional file A3), as well as raw data matrix with anatomical measurements are available online, while general observations are provided below.

**Fig. 3:**
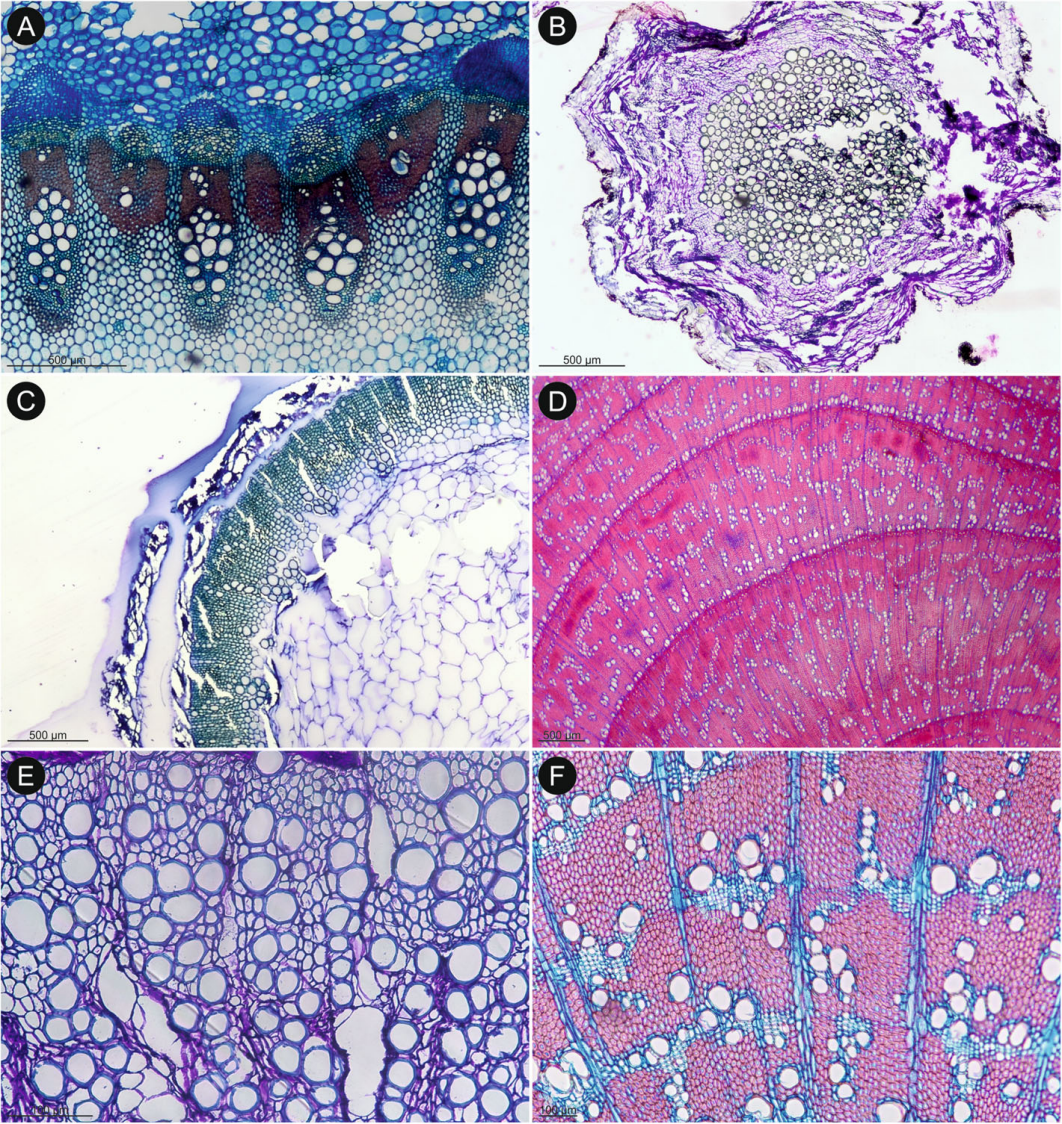
An overview of wood diversity in Tordylieae (**A**-**E**) and outgroup (**F**) in transverse sections. **A** *H. sphondylium*: cambial activity is confined to fascicular zone (within vascular bundles), where cambium-derived fibres are present, interfascicular zones are occupied by medullary rays. **B** *Nanobubon hypogeum*: a section identified as a root due to the missing parenchymatous pith. Cambial activity is best evidenced by wide secondary phloem; the background tissue consists exclusively of parenchyma. **C** *Capnophyllum lutzeyeri*: a continuous, very narrow cylinder of secondary xylem that consists of fibrous background tissue and few vessel elements. **D** *Notobubon pearsonii*: a wide cylinder of fibrous secondary xylem with five growth ring boundaries marked by change in vessel size and production of thick-walled fibres in latewood. **E** *Ducrosia flabellifolia*: the outer part of secondary xylem with exclusively parenchymatous background tissue. **F** *Pycnocycla nodiflora*: fibrous secondary xylem with growth ring boundaries marked by marginal parenchyma and vague dendritic disposition of vessels

**Fig. 4:**
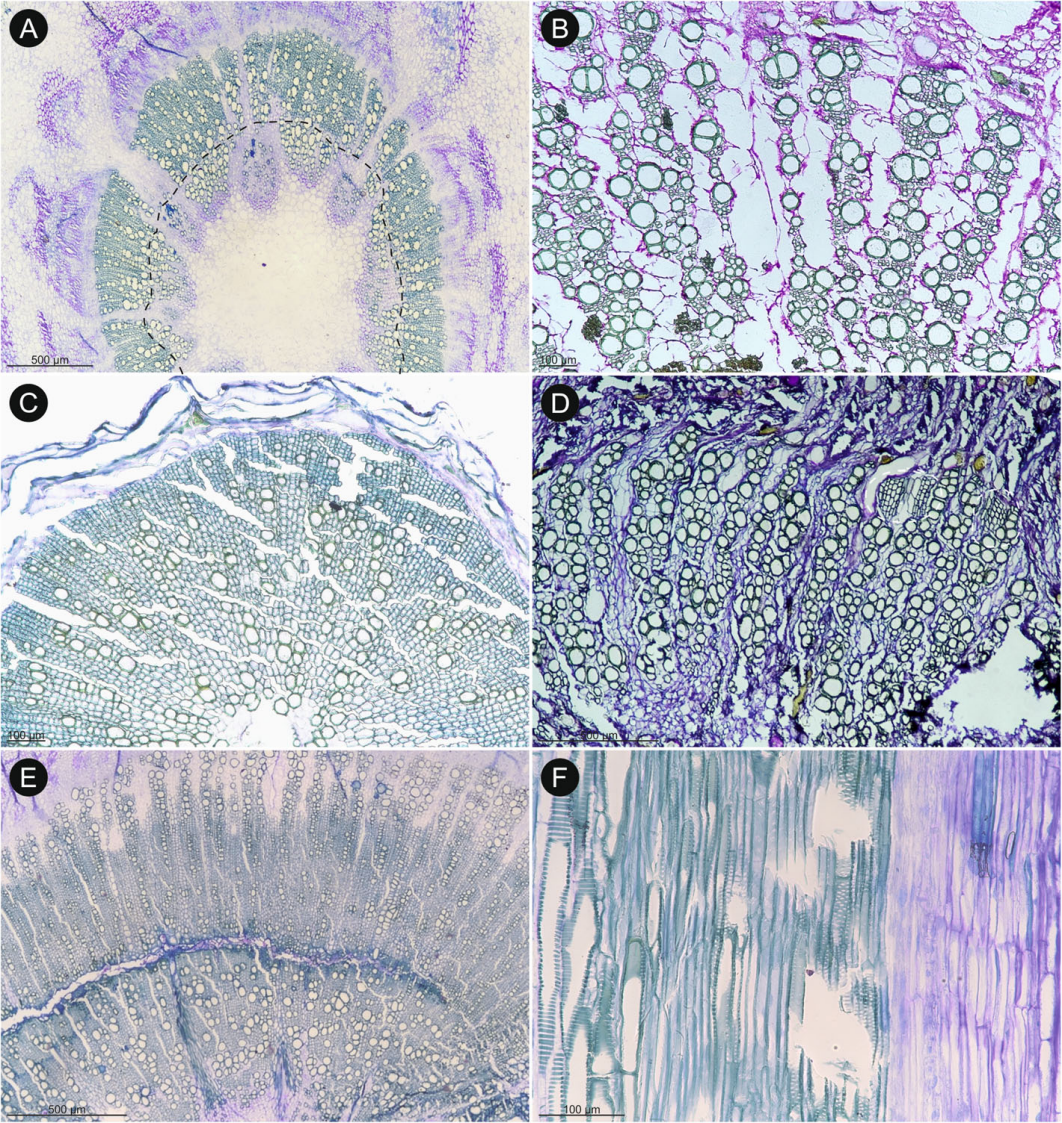
Wood diversity in selected representatives of Tordylieae *Lefebvrea* clade (**A**, **C**–**F**) and subtribe Tordyliinae (**B**). **A** *Capnophyllum macrocarpum* (TS): a considerable amount of secondary xylem and inner, parenchymatous metaxylem (dashed line marks approximate boundary between them). **B** *Semenovia lasiocarpa* (TS): outer portion of secondary xylem with a mixed, patchy type of the background tissue: distorted pervasive parenchyma is dotted with patches of very thick-walled fibres (lower, left corner). The nature of very narrow cells between vessels is unclear – they may be wide fibres or very narrow vessels. **C** *Scaraboides manningii* (TS): section through a root showing exclusively fibrous background tissue. **D** *Cynorhiza typica* (TS): secondary xylem with almost exclusively parenchymatous background tissue: rare patches of thin-walled fibres are encircled with dashed line (upper, right corner). **E** *Dasispermum suffruticosum* (TS): a considerable amount of secondary xylem: the innermost parts of xylem are parenchymatous, while the rest is fibrous. Vessels are disposed indistinctly. **F** *Dasispermum suffruticosum* (RS): transition from vessels with scalariform intervessel pitting (extreme left) to alternate intervessel pitting, axial parenchyma cannot be distinguished from other cells. The bark is stained in violet

**Fig. 5:**
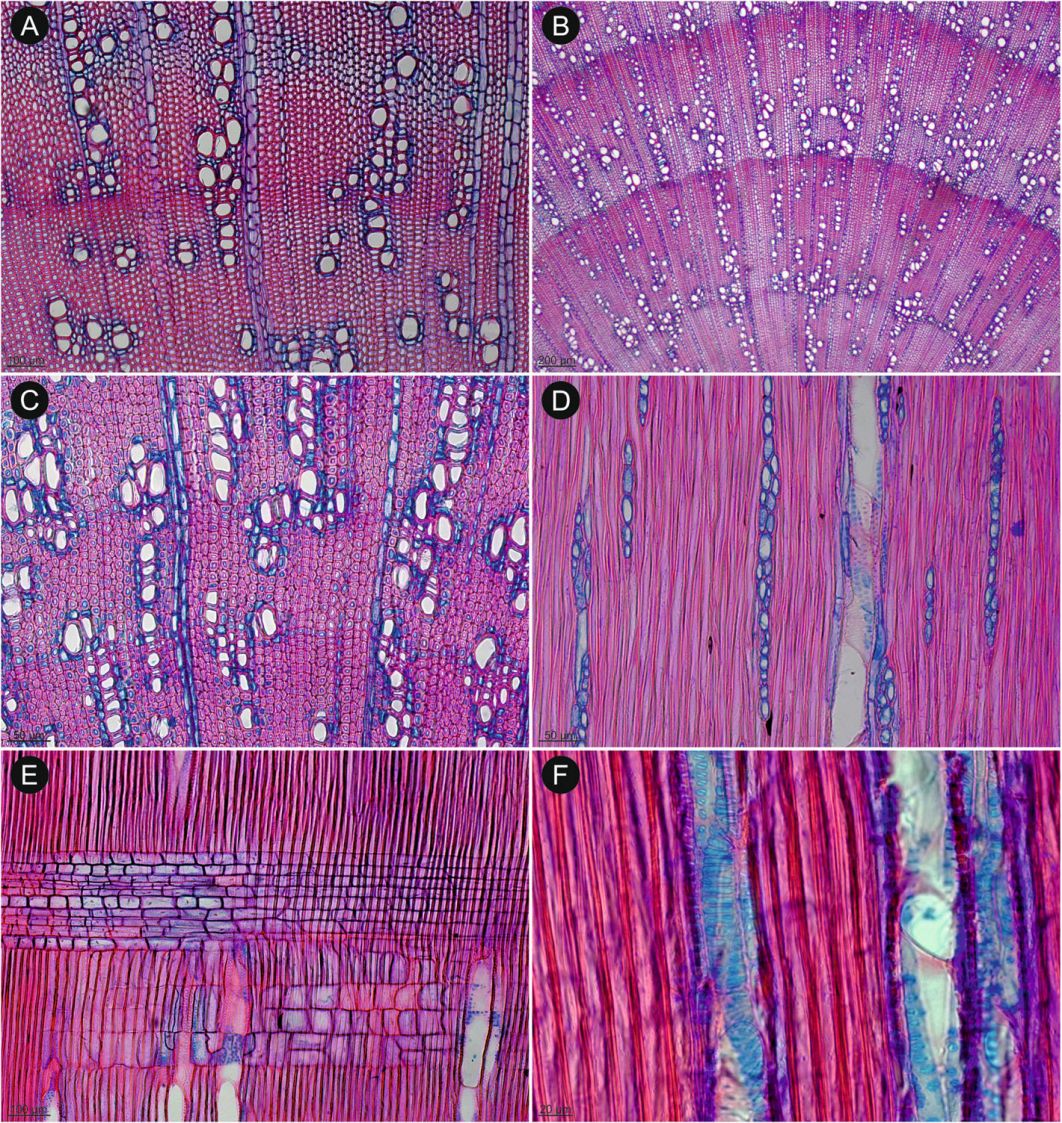
Wood anatomy of selected *Notobubon* species. **A** *Notobubon tenuifolium* (TS): an indistinct growth ring boundary is present in the middle of the photo, marked by layers of radially flattened fibres. **B** *Notobubon capense* (TS): three growth ring boundaries are present (two of them very distinct), marked by change of fibres from moderately thick-walled in earlywood to thick-walled and very thick-walled in latewood, and also by change in vessel diameter. Vessels are concentrated mostly in fascicular regions. **C** *Notobubon striatum* (TS): a diagonal to dendritic vessel disposition, very thick-walled fibres, and paratracheal parenchyma in incomplete sheaths are well-marked in this species. **D** *Notobubon pearsonii* (TLS): uni- and narrow multiseriate rays and intervessel alternate pitting typical of all *Notobubon* species. **E** *Notobubon tenuifolium* (RS): rays composed of exclusively of procumbent (upper), and mixed types of cells (lower), vessel-ray pitting alternate. **F** *Notobubon gummiferum* (RS): simple perforation plate and alternate to scalariform vessel-axial parenchyma pitting

**Fig. 6:**
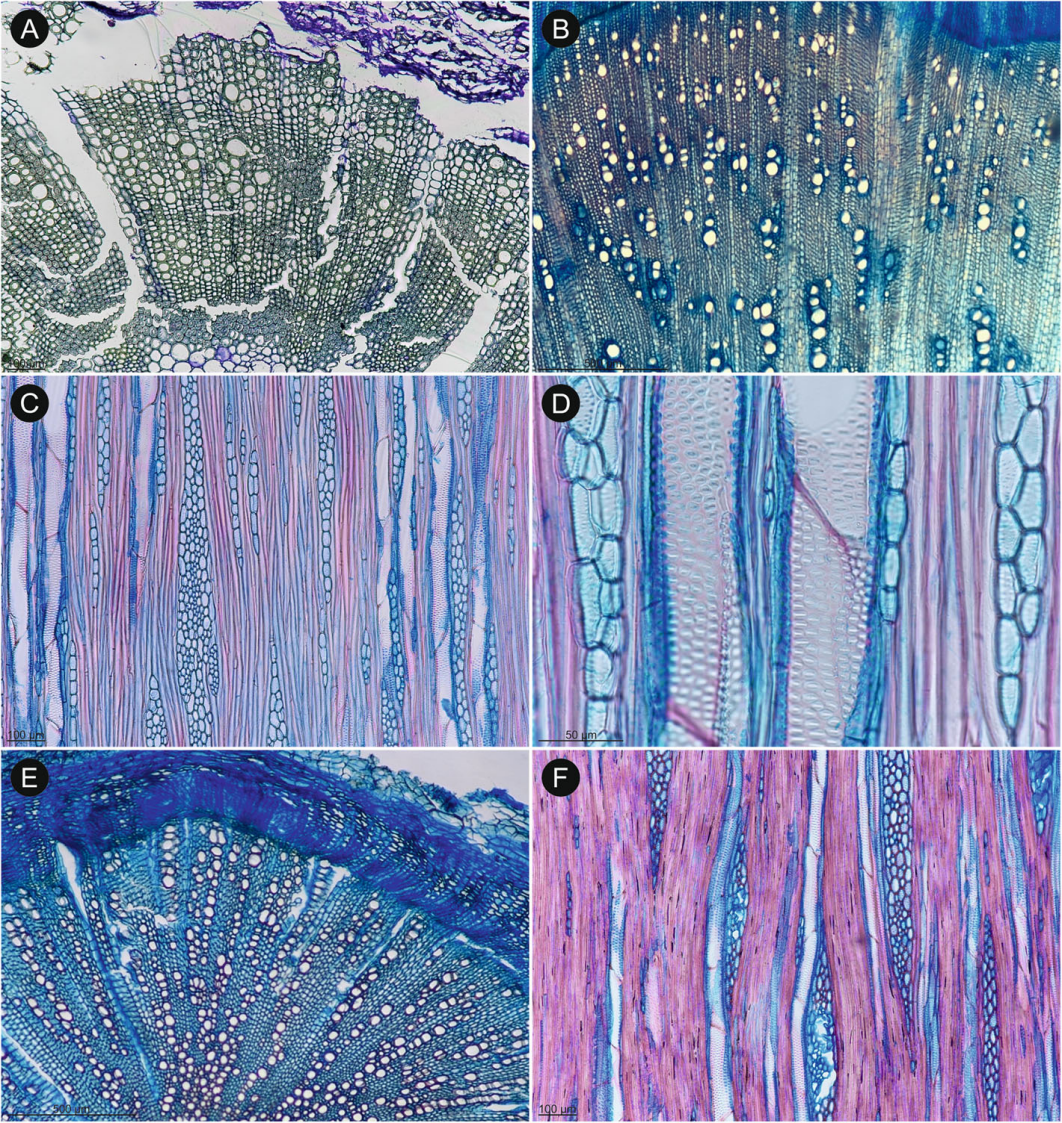
Wood anatomy of selected Tordyliinae (**A**-**E**) and outgroup (**F**). **A** *Stenosemis angustifolia* (TS): exclusively fibrous background tissue composed of moderately thick-walled fibres, wood diffuse-porous, only ray-like structures are bigger cells forming clear uniseriate, radial files. **B** *P. sativa* (TS): diffuse-porous wood, vessels disposed in vague radial pattern, axial parenchyma scanty paratracheal, few multiseriate rays. **C** *P. sativa* (TLS): uni- and multiseriate rays composed mostly of upright and square cells, simple perforation plates. **D** *P. sativa* (TLS): alternate intervessel pitting with pits circular to oval, rounded and with slit-like to narrow lens-like apertures. **E** *Ducrosia anethifolia* (TS): background tissue composed of thin-walled fibres, vessels disposed without clear pattern tending to radial. **F** *Pycnocycla nodiflora* (TLS): multiseriate and few uniseriate rays, vessels with alternate intervessel pitting and simple perforation plates, scanty paratracheal axial parenchyma in strands (best seen along the first vessel from the left)

Our anatomical sampling included a wide range of species: from short-lived therophytes to long-lived phanerophytes (Figs. 3, 4, 5 and 6). In three cases (a sample of *Dasispermum perennans* #2231*, Nanobubon hypogeum*, and *Scaraboides manningii*), the sampled part turned out to be a root rather than a stem (identified by a lack of parenchymatous pith typical for stem; Figs. 3B and 4C). Nevertheless, we decided to include them in the present study as cambial activity in root is most likely paralleled by the production of secondary xylem also in the stem. Thus, even if wood traits observed in a root do not allow to conclude on wood characters in stems, they provide information whether secondary growth is present in the studied species or (almost) completely lost.

The cambial activity was observed in all studied samples irrespectively of their habit or taxonomic relationships (Figs. 3, 4, 5 and 6). In *H. sphondylium* (Fig. 3A), the secondary growth was only initiated, while in the remaining samples, a complete cylinder of secondary xylem was present. It varied in width from less than 1 mm (e.g., *Afrosciadium platycarpum*, *Capnophyllum lutzeyeri*; Fig. 3C) to a few centimetres (particularly in *Notobubon* spp.; Fig. 5; *Capnophyllum macrocarpum*; Fig. 4A). A distinction between fascicular and interfascicular regions was observed in two genera – *Afrosciadium* and selected species of *Notobubon* – where it was marked either by a higher concentration of vessels in fascicular zones or a complete lack of vessels in interfascicular regions. Qualitative wood traits were similar in woody (*Nanobubon*, *Notobubon,* and the outgroup – *Pycnocycla*; Fig. 3D, F) and herbaceous species (the remaining taxa; Fig. 3A–C, E): both groups had wood mostly diffuse-porous and vessels grouped in small clusters and radial multiples with a low percentage of solitary vessels (the least characteristic and the most common vessel grouping in plants [52]). Vessel arrangement was most often indistinct or (vague) radial – a clear juvenile trait. In selected samples of longer-lived, woodier species (*Notobubon*, *Nanobubon*, *Pycnocycla*), a (vague) dendritic pattern was observed further from the pith (Fig. 3D, F). This change from indistinct or radial pattern to dendritic pattern most likely corresponds to a transition from a juvenile to mature wood. Intervessel pitting seems to be (at least weakly) associated with the type of the background tissue: in stems with more abundant axial parenchyma, scalariform pitting was more common, while in stems with more fibres, alternate or transitional forms were dominant.

The background tissue was composed either of fibres or pervasive parenchyma. In most species, it was exclusively fibrous (Figs. 5 and 6). In four samples (*Afrosciadium magalismontanum*, *Cynorhiza typica*, *Ducrosia flabellifolia*, and a root sample of *Nanobubon hypogeum*), the background was exclusively parenchymatous (Figs. 3B,E and 4D). In one species (*Dasispermum suffruticosum*), the inner wood had parenchymatous background and the outer was fibrous (Fig. 4E–F). Additionally, in *Semenovia lasiocarpa* (Fig. 4B), the background was parenchymatous with patches of very thick-walled fibres. The type of the background tissue did not strictly correspond with the habit: stems of woody *Notobubon*, *Nanobubon*, and *Pycnocycla*, and most of the herbaceous species had a fibrous background.

Axial parenchyma was difficult to distinguish in herbaceous plants and, in most cases, it was scanty paratracheal, less often also (almost) vasicentric. Except for the pervasive parenchyma forming the background tissue, it never was very abundant. There are two types of rays: medullary rays are commonly present in most species, while rays of cambial origin are rare and difficult to distinguish from the background. In herbaceous plants, they were clearly observed in few cases.

### Comparison of the ecological niche between woody and herbaceous taxa

**Fig. 7:**
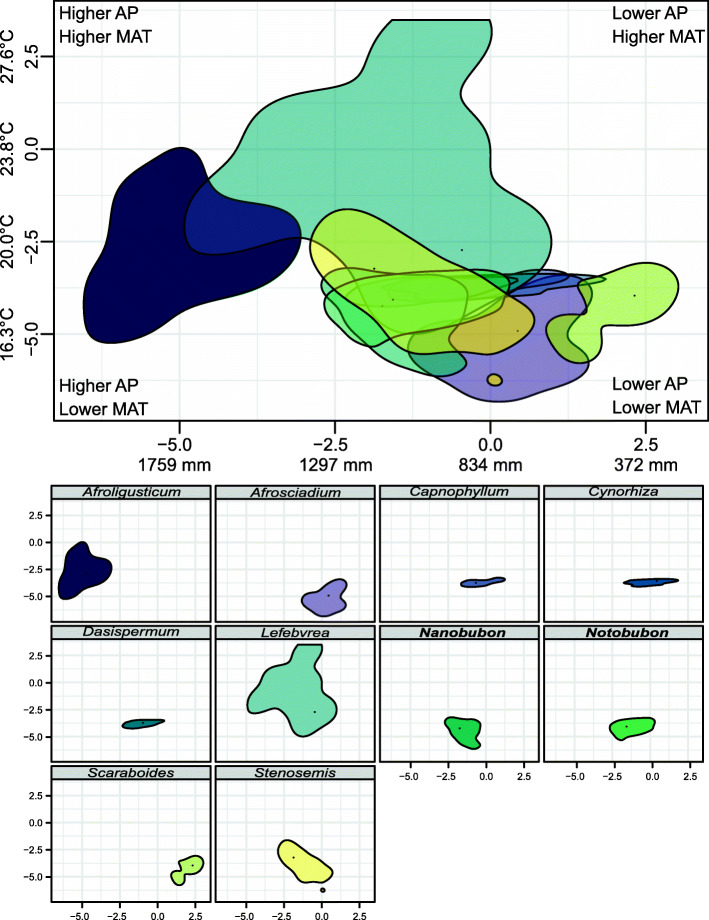
Plots representing climatic niches of the genera from the *Lefebvrea* clade. The abscissa axis shows the first primary component (numbers without units) and the ordinate axis shows the second primary component. The axes were scaled to simultaneously represent change in annual precipitation (AP; abscissa axis) and mean annual temperature (MAT; ordinate axis). Solid contour lines represent 75% of the maximum density estimate for each genus. The top plot presents overlapping of the climatic niches occupied by the *Lefebvrea* clade genera, and smaller plots below represent each genus separately. Colouring in both plots is the same and stands for a given genus. Woody genera are in boldface

Most representatives of the *Lefebvrea* clade occupy ecological niches cooler and less dry than the mean of the African climate space: this is well seen in Fig. 7, where the beginning of the coordinate system (point 0,0 on the plane spanned by the first two principal components) represents the African climatic mean (834 mm of annual precipitation, AP, and 23.8 °C of mean annual temperature, MAT), while most of the taxa fall below this value (i.e., have negative principal component values).

The niches are similar among genera as evidenced by a great degree of their overlapping (top panel in Fig. 7): for most representatives, AP ranges ca. 600–1300 mm, and MAT is between ca. 15–19 °C. The only three clades markedly different from the remainders are *Afroligusticum*, *Lefebvrea*, and *Scaraboides* (Table 1). The first two have broader niches tending to wetter and warmer conditions. The niche of *Afroligusticum* is particularly distinct in its average AP (higher by 366–1313 mm than that of remaining representatives), while *Lefebvrea* typically occupies warmer sites (average MAT higher by 0.7–6.4 °C than other genera; Additional file A4). On the other hand, herbaceous *Scaraboides* is found in niches drier by 208–1313 mm in terms of average AP (the greatest difference is between *Scaraboides* and *Afroligusticum*), but its average MAT is approximately the same as of other genera (Fig. 7, Additional file A4).

**Table 1:** Differences in annual precipitation (lower triangle) and mean annual temperature (upper triangle) between selected African genera of Tordylieae. All comparisons are available online in Additional file A4

	*Afroligusticum*	*Capnophyllum*	*Dasispermum*	*Nanobubon*	*Notobubon*	*Lefebvrea*	*Scaraboides*
*Afroligusticum*	–	1.1–2.9 °C	1.2–3.1 °C	2.2–4.2 °C	2.1–4.0 °C	0.7–3.0 °C	1.2–4.0 °C
*Capnophyllum*	628–854 mm	–	0.0–0.4 °C	0.7–1.6 °C	0.7–1.4 °C	3.2–4.6 °C	0.0–1.7 °C
*Dasispermum*	543–750 mm	27–162 mm	–	0.7–1.5 °C	0.6–1.2 °C	3.3–4.7 °C	0.0–1.5 °C
Nanobubon*	495–712 mm	62–213 mm	0–102 mm	–	0.0–0.6 °C	4.3–5.8 °C	0.0–1.8 °C
Notobubon*	564–768 mm	10–141 mm	0–64 mm	6–119 mm	–	4.2–5.6 °C	0.0–1.6 °C
*Lefebvrea*	366–597 mm	175–345 mm	95–235 mm	44–200 mm	116–253 mm	–	3.2–5.7 °C
*Scaraboides*	1029–1313 mm	311–549 mm	417–633 mm	454–682 mm	393–618 mm	570–810 mm	–

A comparison of 95% confidence intervals for absolute difference in average annual precipitation (lower triangle) and average mean annual temperature (upper triangle) of selected herbaceous and woody genera from the Lefebvrea clade. The intervals were originally expressed in terms of PC1 and PC2 and then back-projected onto AP and MAT dimensions of the bioclimatic space. Taxa with woody habit are marked with asterisks in the first column.

There is almost no difference between the two woody genera, *Nanobubon* and *Notobubon*: their average MAT is virtually the same and average AP is only slightly higher for *Notobubon* (by 6–119 mm). Herbaceous genera are also similar in their preferences for precipitation and temperature (Table 1).

A comparison of woody (*Nanobubon*, *Notobubon*) and herbaceous (the remainders) genera returns striking differences only when the former ones are compared to *Afroligusticum* and *Lefebvrea* (woody taxa occupy places drier by ca. 40–800 mm of average AP and cooler by ca. 2–6 °C of average MAT), and *Scaraboides* (which grows in places drier than woody genera by 393–682 mm of average AP, but with similar average MAT ranges). Otherwise, the niches of woody and herbaceous genera are similar (Fig. 7): *Notobubon* is found in places with similar or only slightly higher (by ≤141 mm) average AP and cooler (by ca. 0.6–1.4 °C of average MAT) than its herbaceous relatives, *Capnophyllum* and *Dasispermum*. The same conclusions are drawn when *Nanobubon* is compared with these two clades.

Generally, the observed climatic differences were greater within the same habit type (e.g., between *Afrosciadium* and *Capnophyllum*; Table 1) than between herbaceous and woody life forms (e.g., *Capnophyllum* and *Notobubon*). Therefore, we found no evidence for a preference of woody genera to occupy markedly drier habitats than their herbaceous relatives. Simultaneously, AP and MAT typical for the *Lefebvrea* clade crown group imply avoidance of extreme temperatures and preference for moderately wet sites. These preferences are, however, shared by woody and herbaceous relatives.

## Discussion

### Climatic preferences of woody and herbaceous taxa

Taken together, herbaceous taxa in our study have a broader niche than their woody cousins (Fig. 7), what is congruent with patterns observed in other clades [53]. This, however, is mostly a result of three genera markedly distinct from the rest: *Afroligusticum*, *Lefebvrea*, and *Scaraboides* (Table 1). Climatic preferences of the first two taxa – tending to wetter and warmer environments (Fig. 7) – reflect the fact that they do not share geographic distribution with most of the *Lefebvrea* clade representatives. Unlike their relatives that are mostly confined to the Cape Floristic Region [54], these two genera span from southern Africa to the Horn of Africa occupying (sub) tropical environments [36, 37]. The lower average MAT of *Afroligusticum* (especially in comparison to *Lefebvrea* of similar geographic distribution) likely results from the fact that this genus grows in Afromontane grasslands and forest habitats [37]. *Afrosciadium* has geographic distribution similar to *Afroligusticum* and *Lefebvrea*, but its niche was resolved as similar to the genera from the Cape Floristic Region (CFR; Fig. 7). This may point to its preference for more moderate climate but it is likely to be an artefact as *Afrosciadium* was represented in our study by only one species (*A. magalismontanum* from southern-African highveld) out of 18 congeners [36].

A comparison of two woody genera (*Nanobubon* and *Notobubon*) shows that their climatic niches are very similar (Fig. 7) and shared with closely related, herbaceous *Capnophyllum* and *Dasispermum* (Table 1). Therefore, we found no evidence for distinct climatic preferences of woody and herbaceous groups. To the contrary, it can be concluded that all studied genera from the Cape Floristic Region occupy similar, moderately wet and cool habitats.

### Woodiness in the Cape Floristic Region

It is not clear how many times the chamaephytic habit evolved in the *Lefebvrea* clade (Fig. 2), but the crown ages of the two woody genera were estimated at 8.49 (10.87–6.22) and 8.85 (11.3–6.42) My ago, that is to the Late Miocene. Unlike wet and mild climates of the Early and Middle Miocene that culminated in the Mid-Miocene Climatic Optimum (18–14 My ago), the Late Miocene experienced a trend towards aridification and high seasonality [55] with the advent of winter rains in the south-western Cape. Simultaneously, most of the radiations within the CFR likely took place sometime in the Late Miocene or around Miocene/Pliocene boundary (ca. 5.3 My ago), when the drying effect of the Benguela current and general climate aridification advanced [54, 56]. Since arid conditions facilitate embolism formation [22], the contemporaneity of climate drying and the evolution of derived woodiness suggests that deposition of a significant amount of xylem, at least in selected CFR clades, may have evolved as a mean counteracting cavitation. The studied genera *Nanobubon* and *Notobubon* fit this scenario.

Interestingly, derived woodiness in other apioid tribes also dates back to the Late Miocene: the arborescent habit of the two carrots from Madeira originated some 7.77 (9.05–6.35) My ago, while shrubby *Billburttia* from Madagascar and *Deverra* found mostly in northern Africa were resolved as 9.0 (10.87–7.14) and 9.86 (11.66–8.08) My old, respectively [42, 43]. Moreover, 80–90% of the derived woody clades from various plant families in the Canary Islands originated within the last 7 My [57]. All of these areas also experienced aridification further supporting the link between the evolution of derived woodiness and drought.

### Herbaceousness in the Cape Floristic Region

It is not clear what was the life form of the last common ancestor of *Capnophyllum*, *Dasispermum*, and *Notobubon* (node C in Fig. 2): in ML analyses, chamaephyte gained the most support but considering that the backbone of the tree was resolved with MP mostly as hemicryptophyte, this habit is also probable. Irrespectively of the precise reconstruction, both methods point to subsequent life span shortening and reduction in body size resulting in an annual therophyte as the last common ancestor of *Capnophyllum* and *Dasispermum* (node D in Fig. 2). Interestingly, this shift must have occurred sometime within (11.63–)9.43–6.26(− 4.92), i.e., at the time of global aridification in the Late Miocene. Therefore, it seems that derived woodiness and annual habit in the *Lefebvrea* clade from the CFR might have evolved as alternative solutions to the same problem of palaeodrought: whereas woody species would cope with aridification through the cavitation-resistant body, the herbaceous taxa would confine their life cycle to wetter seasons, thus avoiding cavitation altogether. This is best mirrored in *Capnophyllum* and *Dasispermum* phenology as they germinate in wet winter, only to wither and die by dry mid-summer [47, 58]. Today, almost 56% of the CFR species are either shrubs or trees; the rest are mostly geophytes [54], thus implying that woody (irrespectively of their ancestral or derived nature) and herbaceous habits are equally suited for periodically dry conditions of the CFR.

It also needs to be borne in mind that the CFR is extremely diversified and embraces a number of distinct vegetation types: from ericoid fynbos, through renosterveld dominated by members of Asteraceae, dry semi-deserts, to coastal plains, and occasional forests [54, 55, 59]. Therefore, even though the woody and herbaceous members of the *Lefebvrea* clade fall into a comparatively narrow and often overlapping range of annual precipitation (ca. 600–1300 mm per year) and mean annual temperature (ca. 15–19 °C), it does not necessarily mean that they inhabit the same microhabitats. For this, however, a more fine-scale analysis is required, especially that a coarse comparison of their habitats based on literature does not return evident dissimilarities [38, 45–47, 58].

### Wood anatomy of southern African umbellifers

Southern Africa harbours four distantly-related linages of woody umbellifers: (1) *Polemanniopsis* and *Steganotaenia* (subfamily Saniculoideae [60]; sometimes treated as tribe Steganotaeniae within Apioideae [36]), (2) *Anginon*, *Glia*, *Heteromorpha*, *Polemannia* (‘*Heteromorpha* clade’, tribe Heteromorpheae, subfamily Apioideae [40]), (3) *Nanobubon* and *Notobubon* (tribe Tordylieae, subfamily Apioideae; this study), and (4) monospecific *Marlothiella* from Namibia. Additionally, three shrubby species of *Deverra* (tribe Apieae, subfamily Apioideae) are found here, but this genus is predominately northern African [43, 44].

Since quantitative wood traits are likely influenced primarily by plant height, we focus here on qualitative characteristics [61, 62]. Although restricted number of available specimens prohibits far-fetched comparisons, our results show that the *Lefebvrea* clade representatives have wood similar to other southern African lineages, despite of being only distantly related. All three clades share a set of characters found commonly in woody apioids (subfamily Apioideae) and saniculoids (subfamily Saniculoideae), like exclusively simple perforation plates and scanty paratracheal axial parenchyma. Growth ring boundaries are either absent (in herbs, e.g., *Afrosciadium*) to present and distinct (often marked by marginal parenchyma and radial flattening of fibres). Wood is most often diffuse-porous, but in longer-lived species with broader cylinder of secondary xylem (*Nanobubon*, *Notobubon* in the present study; *Anginon*, *Polemannia, Heteromorpha* of Heteromorpheae), a tendency towards semi-ring-porosity is present. Solitary vessels are rare and vessel groups are often disposed in radial or (vague) dendritic pattern (the latter particularly in species with broader xylem cylinder). Intervessel pitting ranges from scalariform to alternate and multiple types can be observed in the same species (and specimen). Interestingly, among species examined for this study, helical thickenings were observed in some species of *Notobubon* and *Stenosemis*. This trait is also common in the ‘*Heteromorpha*’ clade [40] but is absent from its closely related Malagasy *Andriana* [63]. Contrarily to earlier studied linages, we observed that in the *Lefebvrea* clade wood ground tissue is composed not only of moderately thick-walled, usually non-septate fibres, but can undergo (at least partial) parenchymatisation. We discuss this trait in the next section.

### Reproductive strategy and the ground tissue

Unlike most other wood traits in Tordylieae, the wood ground tissue shows a wide variation and takes four distinct forms. On one extreme, it is entirely fibrous (e.g., *Notobubon pearsonii*; Fig. 3D), on the other, it consists exclusively of parenchyma (e.g., *Cynorhiza typica*; Fig. 4D). Additionally, at least two distinct intermediate forms are found: zonal (with inner parenchymatous and outer fibrous ground tissue, *Dasispermum suffruticosum*; Fig. 4E) or patchy (ground tissue composed of parenchyma with clusters of fibres, *Semenovia frigida*; Fig. 4B).

In our earlier research [42, 43] on herbaceous and derived woody apioids, we noticed that the entirely fibrous ground tissue was typical for monocarpic taxa irrespectively of their stem architecture, and for polycarpic ones with long internodes, while polycarpic species with shortened internodes (often having leaf rosettes) had parenchymatous ground tissue. This was likely a response to relaxed mechanical requirements of such habit [42, 43]. In other words, the parenchymatisation of ground tissue is seemingly associated with the shortening of internodes, but this effect is probably suppressed by the shift of the shoot or entire plant to the flowering phase presumably affected by the gibberellic acid [42, 64–66]. Since the monocarpic plants typically undergo the shift earlier in their ontogenesis than the polycarpic species, their wood is exclusively fibrous.

Among the species considered for wood anatomy in the present study, the reproductive strategy of one species (*Dasispermum perennans*) is not clear. The fibrous ground tissue is the most common type: it occurs in 27 taxa (*H. sphondylium* with very limited secondary activity, root samples, and mentioned *D. perennans* were excluded). Eight of them are monocarpic (one, *Trigonosciadium viscidulum*, has long internodes, while the rest have shortened internodes). The remaining species are polycarpic and have long internodes, except for *Afrosciadium platycarpum*, which has shortened internodes. Five species (*Afrosciadium magalismontanum*, *Cynorhiza typica*, *Dasispermum suffruticosum*, *Ducrosia flabellifolia*, *Semenovia lasiocarpa*) have parenchymatised wood: all of them are polycarpic and have shortened internodes. Although it must be borne in mind that our sampling is scarce and typically based on one specimen per species, the observed pattern is in good agreement with our hypothesis supporting an interdependence among wood parenchymatisation, internode shortening and polycarpic reproductive strategy.

## Conclusions

All examined Tordylieae members show cambial activity, and wood anatomy is similar in both woody and herbaceous taxa. Wood ground tissue is either fibrous or at least partially parenchymatised: the latter case is often found in polycarpic species with shortened internodes. Therefore, the evolution of derived woodiness in this clade was neither constrained nor stipulated by any particular qualitative wood traits.

Except for the two genera (*Afroligusticum*, *Lefebvrea*), which are distributed from southern Africa to the Horn of Africa, all other members of the *Lefebvrea* clade share similar climatic niches. This parallels their coexistence in the Cape Floristic Region. The origin of the derived woody habit dates back to the Late Miocene: a period of marked climate aridification. The short-lived, annual therophytes that die before the onset of sever summer drought evolved at roughly the same time. This suggests that both habits are alternative solutions to the problem of drought-induced cavitation: herbaceous annuals avoid cavitation altogether through adjustment in timing of their life cycle, while woody taxa withstand it through development of more embolism-resistant bodies.

## Methods

### Molecular analysis

We selected 94 molecular sequences of three markers: nuclear-ribosomal DNA internal transcribed spacer (nrDNA ITS), and plastid *rpoC1*, and *rps16* introns. They represented 53 species: eight species of *Cymbocarpum* clade, seven species of Tordyliineae, 35 species of *Lefebvrea* clade, and three outgroups. Thirty-one sequences were newly obtained, and the rest was retrieved from GenBank. In order to obtain the new sequences, we followed the standard procedure described in detail in our earlier paper [43]. All newly generated sequences were deposited in GenBank (Additional file A1).

Next, we adapted the analytical workflow as described earlier [43]. In brief, we aligned the sequences using E-INS-i algorithm implemented in MAFFT 7.271 [67], manually trimmed primer and partial exon sequences flanking the non-coding regions in Mesquite 3.51 [68], and removed the ambiguously aligned position with the ‘automated1’ algorithm implemented in trimAl 1.2 [69]. Hierarchical clustering of the markers with Concaterpillar 1.7.2 [70] resulted in the concatenation of all three molecular markers, thus we did not detect any topological differences between these datasets; therefore, a concatenated matrix was used in the subsequent analyses (the data matrix with molecular sequences is available online).

### Phylogenetic inference and node calibration

Phylogenetic inference was carried out in two frameworks: maximum likelihood paradigm and Bayesian inference. In both cases, we started by using the BIC metric implemented in the PartitionFinder2 [71] to choose the optimal partitioning scheme and the nucleotide substitution model. For both methods, the sequences were partitioned into ITS and pDNA. In ML as implemented in RAxML, GTR + G model was assigned to both partitions; in BI as implemented in MrBayes, allowing more flexibility in model settings, SYM + G was chosen for ITS, and GTR + G for pDNA. The ML phylogenetic estimation was carried out in RAxML 8.2.4 [72] with branch support estimated based on 1000 rapid bootstrap replicates (ML phylogenetic tree is available online; Additional file A2).

The BI of phylogeny was performed simultaneously with node calibration in MrBayes 3.2.6 [73]. We used three secondary calibration points with posterior probability of 1.0 from an earlier study of subfamily Apioideae [74]. For each of these nodes, the age posterior distribution was evaluated based on a sample of 10,800 phylogenetic trees from that study using treeStat 1.10.0 [75]. Because the posterior distributions did not considerably deviate from the normal distribution as assessed by normal quantile-quantile plots, the calibration points were defined as having normal distributions with mean and standard deviation calculated based on the respective posterior samples. The chosen nodes were defined as the last common ancestor of: (A) *P. sativa* and *Zosima orientalis* (mean age 13.52 Mya with standard deviation of 1.41); (B) *Ducrosia assadii* and *Cymbocarpum wiedemanii* (9.86 Mya ± 1.64); (C) *Dasispermum hispidum* and *Capnophyllum macrocarpum* (5.41 Mya ±1.03); (Fig. 1). We specified the prior probability distribution of branch lengths as uniform, and used the autocorrelated lognormal distribution clock model (‘tk02’). Finally, we applied a relatively non-informative prior assumption (normal distribution with mean = 1, and standard deviation = 5) concerning the base substitution rate of the tree, and set the non-informative uniform prior probability distribution on the root age spanning the range 30–10 My ago [74]. We performed the analysis in two simultaneous runs for 100,000,000 generations each and sampled every 1000 generations, with 25% of the initial samples discarded as a burn-in prior to the calculation of the summary statistics. The results were summarised in the 50% major rule consensus tree. The commands used to perform Bayesian phylogenetic estimation with node calibration are available online.

### Character state evolution

We compiled the data matrix of the reproductive strategy (A, monocarpic; B, polycarpic), life span (A, annual; B, biennial; C, perennial), and life form (A, therophyte; B, hemicryptophyte; C, chamaephyte; D, phanerophyte) for all taxa included in the phylogeny estimation based on the literature [36, 37, 45–47, 58], herbarium specimens, and personal observations (data matrix is available online, Additional file A1). Then, we reconstructed the ancestral states of these traits with two approaches: maximum parsimony and maximum likelihood. For the MP, we employed the 50% majority rule consensus tree from our procedure described in the ‘Phylogenetic inference and node calibration’ section, and the reconstruction was implemented in Mesquite [68]. We chose unordered (Fitch) parsimony for the reproductive strategy and life form, and ordered (Wagner) parsimony for the life span [42, 43].

The ML reconstruction was performed with the MultiState method implemented in BayesTraits 3.0.2 [76]. It allows for the reconstruction of a discrete trait in pre-defined nodes over multiple phylogenetic trees, thus accommodating for the topological uncertainty of the phylogeny. The software also accepts polymorphic states accommodating for the state uncertainty and trait lability. For the ancestral states reconstruction, we took all phylogenetic trees saved from the BI of the phylogeny and used Tracer 1.7.1 [77] to assess when the stationary state was achieved. Based on this, we discarded 10% of the initial samples leaving 180,000 phylogenetic trees. We subsequently used them for ancestral states reconstruction in BayesTraits.

We defined the nodes of interest with AddTag and AddNode commands. In the case of life span, we constrained the possibility of direct transitions between annual and perennial character states reflecting the assumption of ordered parsimony applied in earlier analyses. We summarised the results by counting how many times a given state was resolved with the maximum likelihood in a given node over the 180,000 phylogenetic trees using rank function in R, and by plotting the 95% highest posterior density intervals for all states in a given node with geom_linerange and geom_point functions from the ggplot package in R [78].

While the interpretation of MP results is straightforward, the results of ML will benefit from additional comment. When a trait in a given node is reconstructed with ML, the results take form of a relative likelihood values for each of the states (e.g., 0.7 for the state A vs. 0.3 for the state B). In the case of an iterative reconstruction over multiple trees, one receives multiple relative likelihoods values for each state (they differ because the topology and/or branch lengths of each tree are slightly different affecting the reconstruction). To summarise these results, one can simply count how many times a given state was selected over the other state (i.e., how many times a given state ‘won’). However, it is possible that even when the same state is reconstructed 100% times in a given node, its relative likelihood may be only minimally higher than the likelihood for the other state (e.g., 0.51 for the state A vs. 0.49 for the state B). In other words, the number of winnings does not provide any information about how strong the victory was. Therefore, while interpreting our results, we paid attention to the number of winnings (summarised as percentage values in the second plate of Fig. 2) and to the 95% highest posterior density intervals (presented as plots in Fig. 2).

### Wood anatomical analysis

We considered 39 species represented by 49 specimens for wood anatomical characters. The samples were obtained from herbarium collections (JRAU, NBG; acronyms follow [79]), with the exception of *H. sphondylium* #2059 and *P. sativa* #2056 that were gathered from the wild and identified by Łukasz Banasiak (no collection permissions were required). Voucher data for all specimens are available online (Additional file A1). Our sampling was primarily focused on the *Lefebvrea* clade (29 species, 37 specimens), but also included other Tordylieae (seven species, seven specimens) and the woody outgroup (*Pycnocycla*, Echinophoreae: three species, five specimens). Wood samples were taken from the base of the main stem or thick lateral branches of living specimens or from the thickest, usually basal-most, part of the preserved herbarium specimen. Fresh material was immediately stored in 70% ethanol.

Samples were sectioned and stained following the standard procedures described elsewhere [42]. Wood anatomical characters were examined with light microscopy and wood descriptions followed the International Association of Wood Anatomists’ list of microscopic features for hardwood identification whenever possible [52]. Although care was taken for the quantitative traits to be assessed correctly, it should be kept in mind that measuring certain traits in samples with very limited secondary growth may be tricky: e.g., obtaining straight tangential longitudinal sections necessary for correct estimation of ray number per mm may be impossible and assessing the number of vessels per sq. mm may be biased due to the clustered disposition of vessels in juvenile wood. Additionally, in herbaceous species, it was often difficult to distinguish between very narrow vessels and wider fibres – a problem also observed in other clades [80] – and therefore tangential vessel diameters may be somewhat underestimated. Length of the tracheary elements was measured after maceration as described in [81].

### Estimation of the ecological niche

We decided to perform the ecological niche estimation at the generic level. This was dictated by a number of reasons: (1) species distributions observed in the available databases represent their realized niches affected by multitude of factors that are of no interest in the present study (e.g., human activity, sampling bias); (2) if congeners share at least moderate niche conservatism, aggregating them and estimating the ecological niche at the generic level will result in an estimation that is more similar to the fundamental niche than to the realized one. Finally, (3) the number of available, high quality occurrence data points for particular species was low, rendering the estimation at species level unreliable.

All analysed data come from a spatial extent defined by four pairs of coordinates (20.0 N 0.0E; 20.0 N 50.0E; 35.0S 0.0E, 35.0S 50.0E) covering the whole area, where the *Lefebvrea* clade members naturally occur. For the species included in the phylogenetic analysis, we retrieved from the GBIF only those specimen occurrences that had radius of uncertainty ≤50 km [75]. The query was executed using occ_search command from R package rgbif [77]. Next, we removed duplicated records mirrored from the SANBI Plants of southern Africa database, which was accessed independently using online tools [76]. In the subsequent analysis, each of the specimen occurrences was represented by a single, randomly-chosen point from within the uncertainty radius. Then, we retrieved the bioclimatic variables from the WorldClim 2.1 database with 2.5 arc minute spatial resolution [78] and performed the Primary Component Analysis (PCA) on these variables for the full study extent to accommodate for strong correlations between raw variables as some of them are calculated based on the others. The PCA was executed with rasterPCA command from the raster package in R [79]. The two first principal components (PC) explained 44.9 and 28.3% of the total variance, respectively. The first principal component (PC1) was positively correlated with higher temperature seasonality and amplitude and negatively correlated with isothermality and higher annual precipitation (AP). The second principal component (PC2) was clearly positively correlated with mean annual temperature (MAT; the results of PCA are available online).

Next, we estimated the ecological niche of each genus employing two-dimensional kernel density estimation, where one dimension represented the first principal component (PC1) and the other represented the second principal component (PC2). This was done with MASS package and visualised with ggplot2 package in R [71, 80]. We also calculated the 95% joint confidence intervals for the difference of means in PC1 and PC2 between pairs of each genera. This was performed with T3 procedure that is a generalised version of Tukey-Kramer procedure [81] with DTK.test command in DTK package [82]. Finally, to facilitate the interpretation, we back-projected PC1 and PC2 onto the annual precipitation (AP) and mean annual temperature (MAT) dimensions of the bioclimatic space. AP and MAT constitute two main descriptors of the climate that were strongly correlated with the first two principal components in our analyses. The plots showing comparisons between all genera are available online (Additional file A4).

## Supplementary Information

**Additional file 1.** Accession table of specimens used in this study.

**Additional file 2.** Maximum likelihood phylogenetic trees with bootstrap values.

**Additional file 3.** Detailed wood descriptions.

**Additional file 4.** Plots of 95% confidence intervals for the difference of means in the first (PC1) and the second (PC2) primary components back-projected onto average mean annual precipitation and temperature dimensions of the bioclimatic space between pairs of genera.

## Data Availability

The datasets generated and analysed during the current study are available in the FigShare repository, DOI: 10.6084/m9.figshare.13026935 and consist of: (1) a matrix with molecular sequences before and after trimming, (2) the command file employed for BI of phylogeny with node calibration, (3) the molecular matrix necessary for replication of the BI, (4) the dated 50% majority rule consensus tree from Bayesian analysis, (5) matrix with reproductive strategy, life span, and life form states, (6) the results of PCA, (7) a matrix with quantitative wood traits measurements.

## Abbreviations

AP: annual precipitation
BI: Bayesian inference
BS: bootstrap support
CDN: a clade of *Capnophyllum*, *Dasispermum* (including *Scaraboides*), and *Notobubon* (excluding *N. pearsonii*)
CFR: Cape Floristic Region
HPD: highest posterior density
MAT: mean annual temperature
MCMC: Markov Chain Monte Carlo
ML: maximum likelihood
MP: maximum parsimony
PC: principal component
PCA: principal component analysis
pDNA: plastid DNA
PP: posterior probability
RS: radial section
TLS: tangential longitudinal section
TS: transverse section

## References

[1] Doyle JA (2012). Molecular and fossil evidence on the origin of angiosperms. Annu Rev Earth Planet Sci.

[2] Carlquist S (2013). More woodiness/less woodiness: evolutionary avenues, ontogenetic mechanisms. Int J Plant Sci.

[3] Feild TS, Arens NC, Doyle JA, Dawson TE, Donoghue MJ. Dark and disturbed: a new image of early angiosperm ecology. Paleobiology. 2004;30(1):82–107. 10.1666/0094-8373(2004)030<0082:DADANI>2.0.CO;2.

[4] Schweingruber F, Landolt W. The Xylem Database. Swiss Federal Institute for Forest, Snow and Landscape Research https://www.wsl.ch/dendropro/xylemdb/. 2010. Accessed 28 Sep 2020.

[5] Sinnott EW, Bailey IW (1915). The evolution of herbaceous plants and its bearing on certain problems of geology and climatology. J Geol.

[6] Dulin MW, Kirchoff BK (2010). Paedomorphosis, secondary woodiness, and insular woodiness in plants. Bot Rev.

[7] Lens F, Davin N, Smets E, del Arco M (2013). Insular woodiness on the Canary Islands: a remarkable case of convergent evolution. Int J Plant Sci.

[8] Schweingruber FH, Börner A, Schulze E-D. Atlas of stem anatomy in herbs, shrubs and trees volume I. 1st ed. Heidelberg, New York, Dordrecht, London; 2013. 10.1007/978-3-642-11638-4.

[9] Schweingruber FH, Börner A, Schulze E-D (2013). Atlas of stem anatomy in herbs, shrubs and trees volume II.

[10] Kidner C, Groover A, Thomas DC, Emelianova K, Soliz-Gamboa C, Lens F. First steps in studying the origins of secondary woodiness in *Begonia* (Begoniaceae): combining anatomy, phylogenetics, and stem transcriptomics. Biol J Linn Soc. 2015;117:121–38. 10.1111/bij.12492.

[11] Rowe N, Paul-Victor C (2012). Herbs and secondary woodiness-keeping up the cambial habit. New Phytol.

[12] Roodt D, Li Z, Van De Peer Y, Mizrachi E, Gaut B (2019). Loss of wood formation genes in monocot genomes. Genome Biol Evol.

[13] Jura-Morawiec J, Tulik M (2010). Budowa pni drzew jednoliściennych Stem structure of monocotyledonous trees. Sylwan..

[14] Jura-Morawiec J, Tulik M, Iqbal M (2015). Lateral meristems responsible for secondary growth of the monocotyledons: a survey of the state of the art. Bot Rev.

[15] Lens F, Smets E, Melzer S (2012). Stem anatomy supports *Arabidopsis thaliana* as a model for insular woodiness. New Phytol.

[16] Melzer S, Lens F, Gennen J, Vanneste S, Rohde A, Beeckman T (2008). Flowering-time genes modulate meristem determinacy and growth form in *Arabidopsis thaliana*. Nat Genet.

[17] Carlquist S (1974). Island biology.

[18] Caujapé-Castells J, Tye A, Crawford DJ, Santos-Guerra A, Sakai A, Beaver K, Lobin W, Vincent Florens FB, Moura M, Jardim R (2010). Conservation of oceanic island floras: present and future global challenges. Perspect Plant Ecol Evol Syst.

[19] Merckx VSFT, Hendriks KP, Beentjes KK, Mennes CB, Becking LE, Peijnenburg KTCA, Afendy A, Arumugam N, de Boer H, Biun A, Buang MM, Chen PP, Chung AYC, Dow R, Feijen FAA, Feijen H, Soest CFV, Geml J, Geurts R, Gravendeel B, Hovenkamp P, Imbun P, Ipor I, Janssens SB, Jocqué M, Kappes H, Khoo E, Koomen P, Lens F, Majapun RJ, Morgado LN, Neupane S, Nieser N, Pereira JT, Rahman H, Sabran S, Sawang A, Schwallier RM, Shim PS, Smit H, Sol N, Spait M, Stech M, Stokvis F, Sugau JB, Suleiman M, Sumail S, Thomas DC, van Tol J, Tuh FYY, Yahya BE, Nais J, Repin R, Lakim M, Schilthuizen M (2015). Evolution of endemism on a young tropical mountain. Nature..

[20] Neupane S, Lewis PO, Dessein S, Shanks H, Paudyal S, Lens F (2017). Evolution of woody life form on tropical mountains in the tribe Spermacoceae (Rubiaceae). Am J Bot.

[21] Nürk NM, Atchison GW, Hughes CE (2019). Island woodiness underpins accelerated disparification in plant radiations. New Phytol.

[22] Lens F, Tixier A, Cochard H, Sperry JS, Jansen S, Herbette S (2013). Embolism resistance as a key mechanism to understand adaptive plant strategies. Curr Opin Plant Biol.

[23] Francisco-Ortega J, Santos-Guerra A, Hines A, Jansen RK (1997). Molecular evidence for a mediterranean origin of the macaronesian endemic genus *Argyranthemum* (Asteraceae). Am J Bot.

[24] Dória LC, Podadera DS, del Arco M, Chauvin T, Smets E, Delzon S (2018). Insular woody daisies (*Argyranthemum*, Asteraceae) are more resistant to drought-induced hydraulic failure than their herbaceous relatives. Funct Ecol.

[25] Tixier A, Cochard H, Badel E, Dusotoit-Coucaud A, Jansen S, Herbette S (2013). *Arabidopsis thaliana* as a model species for xylem hydraulics: does size matter?. J Exp Bot.

[26] Dória LC, Meijs C, Podadera DS, del Arco M, Smets E, Delzon S, Lens F (2019). Embolism resistance in stems of herbaceous Brassicaceae and Asteraceae is linked to differences in woodiness and precipitation. Ann Bot.

[27] Lens F, Groeninckx I, Smets E, Dessein S (2009). Woodiness within the Spermacoceae-Knoxieae alliance (Rubiaceae): retention of the basal woody condition in Rubiaceae or recent innovation?. Ann Bot.

[28] Lens F, Eeckhout S, Zwartjes R, Smets E, Janssens SB (2012). The multiple fuzzy origins of woodiness within Balsaminaceae using an integrated approach. Where do we draw the line?. Ann Bot.

[29] Lens F, Picon-Cochard C, EL Delmas C, Signarbieux C, Buttler A, Cochard H (2016). Herbaceous angiosperms are not more vulnerable to drought-induced embolism than angiosperm trees. Plant Physiol.

[30] Carlquist S (1997). Wood anatomy of *Argyroxiphium* (Asteraceae): adaptive radiation and ecological correlations. J Torrey Bot Soc.

[31] Darwin C (1859). On the origin of species by means of natural selection, or, the preservation of favoured races in the struggle for life.

[32] Wallace AR (1878). Tropical nature, and other essays.

[33] Böhle U-R, Hilger HH, Martin WF (1996). Island colonization and evolution of the insular woody habit in *Echium* L. (Boraginaceae). Proc Natl Acad Sci U S A.

[34] Carlquist S (2017). Vestured pits in *Echium* (Boraginaceae): island woodiness revisited. Aliso..

[35] Plunkett GM, Pimenov MG, Reduron J-P, Kljuykov EV, Lee B-Y, Van Wyk B-E, Kadereit JW, Bittrich V, Kubitzky K (2018). Apiaceae. The families and genera of vascular plants. Volume XV. Flowering plants. Eudicots: Apiales, Gentianales (except Rubiaceae).

[36] Van Wyk B-E, Tilney PM, Magee AR. African Apiaceae: a synopsis of the Apiaceae/Umbelliferae of sub-Saharan Africa and Madagascar. 1st ed. Pretoria: Briza Academic Books; 2013.

[37] Winter PJD, Magee AR, Phephu N, Tilney PM, Downie SR, Van Wyk B-E (2008). A new generic classification for African peucedanoid species (Apiaceae). Taxon..

[38] Magee AR, van Wyk BE, Tiney PM, Downie SR (2009). Generic delimitations and relationships of the cape genera *Capnophyllum*, *Dasispermum*, and *Sonderina*, the north African genera *Krubera* and *Stoibrax*, and a new monotypic genus of the subfamily Apioideae (Apiaceae). Syst Bot.

[39] Magee AR, Calviño CI, Liu M, Downie SR, Tilney PM, van Wyk BE (2010). New tribal delimitations for the early diverging lineages of Apiaceae subfamily Apioideae. Taxon..

[40] Oskolski AA, Van Wyk BE (2008). Systematic and phylogenetic value of wood anatomy in Heteromorpheae (Apiaceae, Apioideae). Bot J Linn Soc.

[41] Stepanova AV, Oskolski AA (2010). Wood anatomy of *Bupleurum* L. (Apioideae, Apiaceae) in relation to habit, phylogenetic relationships, and infrageneric taxonomy. Plant Divers Evol.

[42] Frankiewicz KE, Oskolski AA, Banasiak Ł, Fernandes F, Reduron J-P, Reyes-Betancort JA, Szczeparska L, Alsarraf M, Baczyński J, Spalik K (2020). Parallel evolution of arborescent carrots (Daucus) in Macaronesia. Am J Bot.

[43] Frankiewicz KE, Banasiak Ł, Oskolski AA, Reduron J-P, Reyes-Betancort JA, Alsarraf M, et al. Long-distance dispersal events rather than growth habit and life-history traits affect diversification rate in tribe Apieae (Apiaceae). Bot J Linn Soc. 2021; In press. 10.1093/botlinnean/boab032.

[44] van Munster S, Magee AR, Zietsman PC (2019). *Deverra rapaletsa* (Apiaceae), a new limestone endemic species from the Ghaap plateau, northern cape, South Africa. South African J Bot.

[45] Magee AR, Van Wyk B-E, Tilney PM, Botany SS, Mar NJ, Magee AR (2009). A taxonomic revision of the woody south African genus *Notobubon* (Apiaceae: Apioideae). Syst Bot.

[46] Magee AR, Van Wyk B-E, Tilney PM (2008). A taxonomic revision of the genus *Nanobubon* (Apiaceae: Apioideae). South African J Bot.

[47] Magee AR, Van Wyk B-E, Tilney PM, Downie SR (2010). A taxonomic revision of the south African endemic genus *Dasispermum* (Apiaceae, Apioideae). South African J Bot.

[48] Press JR, Dias E (1998). The genera *Melanoselinum* Hoffm. And *Angelica* L. (Umbelliferae) in Macaronesia. Arquipélago Life Mar Sci.

[49] Pimenov MG, Degtjareva GV, Ostroumova TA, Samigullin TH, Averyanov LV (2016). Xyloselinum laoticum (Umbelliferae), a new species from Laos, and taxonomic placement of the genus in the light of nrDNA ITS sequence analysis. Phytotaxa..

[50] Downie SR, Spalik K, Katz-Downie DS, Reduron J-P (2010). Major clades within Apiaceae subfamily Apioideae as inferred by phylogenetic analysis of nrDNA ITS sequences. Plant Divers Evol.

[51] Raunkiaer C (1934). The life forms of plants and statistical geography: being the collected papers of C. Raunkiaer.

[52] IAWA Committee (1989). IAWA list of microscopic features for hardwood identification with an appendix on non-anatomial information. IAWA Bull.

[53] Smith SA, Beaulieu JM (2009). Life history influences rates of climatic niche evolution in flowering plants. Proc R Soc B Biol Sci.

[54] Goldblatt P, Manning JC (2002). Plant diversity of the cape region of southern Africa. Ann Missouri Bot Gard.

[55] Linder HP (2005). Evolution of diversity: the cape flora. Trends Plant Sci.

[56] Linder HP (2003). The radiation of the cape flora, southern Africa. Biol Rev Camb Philos Soc.

[57] Huysduynen AH van, Janssens S, Merckx V, Vos R, Valente L, Zizka A, et al. Multiple origins of insular woodiness on the Canary Islands are consistent with palaeoclimatic aridification. bioRxiv. 2020. 10.1101/2020.05.09.084582.

[58] Magee AR, van Wyk BE, Tilney PM, Downie SR (2009). A taxonomic revision of *Capnophyllum* (Apiaceae: Apioideae). South African J Bot.

[59] Linder HP (2001). Plant diversity and endemism in sub-Saharan tropical Africa. J Biogeogr.

[60] Oskolski AA, Rossouw AS, Van Wyk BE (2010). Wood and bark anatomy of *Steganotaenia* and *Polemanniopsis* (tribe Steganotaenieae, Apiaceae) with notes on phylogenetic implications. Bot J Linn Soc.

[61] Olson ME, Anfodillo T, Gleason SM, McCulloh KA (2021). Tip-to-base xylem conduit widening as an adaptation: causes, consequences, and empirical priorities. New Phytol.

[62] Frankiewicz KE, Chau JH, Oskolski AA (2021). Wood and bark of *Buddleja*: uniseriate phellem, and systematic and ecological patterns. IAWA J.

[63] Long C, Oskolski AA (2018). Wood and bark anatomy of *Andriana* (Heteromorpheae, Apiaceae) with phylogenetic and ecological implications. South African J Bot.

[64] Conti L (2017). Hormonal control of the floral transition: can one catch them all?. Dev Biol.

[65] Ragni L, Nieminen K, Pacheco-Villalobos D, Sibout R, Schwechheimer C, Hardtke CS (2011). Mobile gibberellin directly stimulates *Arabidopsis* hypocotyl xylem expansion. Plant Cell.

[66] Sibout R, Plantegenet S, Hardtke CS (2008). Flowering as a condition for xylem expansion in *Arabidopsis* hypocotyl and root. Curr Biol.

[67] Katoh K, Standley DM (2013). MAFFT multiple sequence alignment software version 7: improvements in performance and usability. Mol Biol Evol.

[68] Maddison WP, Maddison DR. Mesquite: a modular system for evolutionary analysis. http://www.mesquiteproject.org. 2019. Accessed 20 Sep 2020.

[69] Capella-Gutiérrez S, Silla-Martínez JM, Gabaldón T (2009). trimAl: a tool for automated alignment trimming in large-scale phylogenetic analyses. Bioinformatics.

[70] Leigh JW, Susko E, Baumgartner M, Roger AJ (2008). Testing congruence in phylogenomic analysis. Syst Biol.

[71] Lanfear R, Calcott B, Ho SYW, Guindon S (2012). PartitionFinder: combined selection of partitioning schemes and substitution models for phylogenetic analyses. Mol Biol Evol.

[72] Stamatakis A (2014). RAxML version 8: a tool for phylogenetic analysis and post-analysis of large phylogenies. Bioinformatics..

[73] Ronquist F, Teslenko M, Van Der Mark P, Ayres DL, Darling A, Höhna S (2012). Mrbayes 3.2: efficient bayesian phylogenetic inference and model choice across a large model space. Syst Biol.

[74] Banasiak Ł, Piwczyński M, Uliński T, Downie SR, Watson MF, Shakya B, Spalik K (2013). Dispersal patterns in space and time: a case study of Apiaceae subfamily Apioideae. J Biogeogr.

[75] Rambaut A, Drummond AJ (2018). TreeStat tree statistic calculation tool.

[76] Pagel M, Meade A (2019). BayesTraits.

[77] Rambaut A, Drummond AJ, Xie D, Baele G, Suchard MA (2018). Posterior summarization in Bayesian phylogenetics using tracer 1.7. Syst Biol.

[78] Wickham H (2009). Ggplot2: elegant graphics for data analysis.

[79] Thiers BM. (continously updated) Index Herbariorum: A global directory of public herbaria and associated staff: New York Botanical Garden’s Virtual Herbarium. http://sweetgum.nybg.org/ih/; 2013.

[80] Lens F, Endress ME, Baas P, Jansen S, Smets E (2009). Vessel grouping patterns in subfamilies Apocynoideae and Periplocoideae confirm phylogenetic value of wood structure within Apocynaceae. Am J Bot.

[81] Franklin GL (1945). Preparation of thin sections of synthetic resins and wood-resin composites, and a new macerating method for wood. Nature..

[82] Lau M. R package: Dunnett-Tukey-Kramer pairwise multiple comparison test adjusted for unequal variances and unequal sample sizes. 2013. https://cran.r-project.org/web/packages/DTK/DTK.pdf. Accessed 28 Sep 2020.

