# Supplementary material for: Derived woodiness and annual habit evolved in African umbellifers as alternative solutions for coping with drought
Source: BMC Plant Biol. 2021 Aug 20;21:383. doi: 10.1186/s12870-021-03151-x (PMC8377965; doi:10.1186/s12870-021-03151-x)
Supplement: Supplementary file 1 — Additional file 1. Accession table of specimens used in this study. [file 12870_2021_3151_MOESM1_ESM.pdf]

Additional file A1. Accessions of tribe Tordylieae and species used as outgroups with corresponding GenBank reference numbers. The accessions used to obtain new sequences and these considered for wood anatomy are provided with detailed voucher information and accession ID, while accessions retrieved from GenBank are provided only with taxonomic information and GenBank reference numbers. All herbarium acronyms follow Index Herbariorum [1]. The letter 'A' following accession ID denotes specimen used in the anatomical study.

**Taxon**	**Accession ID**	**Voucher information**	**GenBank accession numbers**		
			ITS	*rpoC1* intron	*rps16* intron
		**Sequences newly obtained and specimens considered for wood anatomy**			
**Outgroup**					
Echinophora chrysantha Freyn & Sint.	#0746	Turkey, Erzincan Prov., Kemah-Refahiye Rd., 10 km north of Kemah, 1250 m, 17 July 1988, *M.Nydegger 43808* (KRAM 436832)			MW166381
Echinophora chrysantha Freyn & Sint.	#0842	Turkey, Erzincan Prov., about 10 km north-west of Erzincan by road, 1500 m, 2 August 1978, *F.Ehrendorfer, F.Sorger, D.Fürnkranz, M.A.Fischer, A.Öztürk 787-166-1* (WU)		MW166374	
Pycnocycla aucheriana Boiss.	#1713 A	Iran, Balochistan, 64 km north-west from Bazman, road to Bam, 1000 m, 5 May 1977, *K.H.Rechinger* (E 00787234)			
Pycnocycla aucheriana Boiss.	#1715 A	Pakistan, Balochistan, ca 15 km east of Khuzdar towards Zeedi (Zidi) and Larkana, 1160 m, 8 May 1990, *A.Gzhofoor & Steve M. Goodman 4958* (E 00787273)			
Pycnocycla caespitosa Boiss. & Hausskn	#1714 A	Iran, Kohgilouyeh-Boirahmad, 16km from Dogonbadan to Choram, near Deel (Dil), 1500–1900 m, 23 July 1983, *Assadi & Abouhamzeh 46578* (E 00787244)	MW166363		MW166382
Pycnocycla caespitosa Boiss. & Hausskn	#2145 A	Iran, Daleki (Dalaki), 26 April 1885, *O.Stapf 557* (WU 0095731)			
Pycnocycla nodiflora Decne. ex Boiss.	#1716 A	Iran, Kerman province, Aliabad to Esfandaqeh (Esfandagheh), 122 km east of Aliabad, at Ab-e Dasht, 1650 m, 11 June 1977, *Assadi, Edmondson & Miller 1948* (E 00787258)	MW166362	MW166375	
***Cymbocarpum* clade**					
Cymbocarpum erythraeum (DC.) Boiss.	#0937	Turkey, Erzurum Prov., Palandöken Mt, directly south of Erzurum, hillside road near ruins of monastery in proximity to antenna station located at the peak, ca 2900 m, 26 July 1978, *F.Ehrendorfer, F.Sorger, D.Fürnkranz & M.A.Fischer 787-114-2* (WU)	MW166352	MW166364	MW166376
Ducrosia anethifolia Boiss.	#1721 A	United Arab Emirates, Abu Dhabi emirate, Al Ayn. UTM ref. CB 7477, 300 m, 16 February 1980, *J.R.Edmondson 3013* (E 00648772)			
Ducrosia anethifolia Boiss.	#2132	Iran, Schiraz, 16 June 1885, *O.Stapf 1455* (WU 0095742)	MW166361	MW166373	
Ducrosia flabellifolia Boiss.	#1720 A	Saudi Arabia, volcano west of Al Ays, 3000 ft, 3 April 1989, *I.S.Collenette 7072* (E 00648778)			
***Lefebvrea* clade**					
Afrosciadium magalismontanum (Sond.) P.J.D.Winter	#1917 A	South Africa, Johannesburg, Melville Koppies nature reserve, 14 June 2016 (ethanol-preserved collection at the Department of Botany and Plant Biotechnology, University of Johannesburg, Johannesburg, South Africa)			
Afrosciadium platycarpum (Sond.) P.J.D.Winter	#2211 A	South Africa, St. Bernands, 21 December 1952, *W.Z.Barker 7954* (NBG 22668)			
Capnophyllum africanum (L.) Gaertn.	#2242 A	South Africa, along R27, from Velddrif to Cape Town, *Boatwright et al. 228* (JRAU JSB228)	MW166357	MW166371	MW166379
Capnophyllum leiocarpon (Sond.) J.C.Manning & Goldblatt	#2216 A	South Africa, Western Cape, 3218 DC, 16 km west of Piketberg, Zuurfontein 139, 75 m, 30 September 2010, *N.A.Helme 6778* (NBG 0277988-0)		MW166370	
Capnophyllum lutzeyeri Magee & B.-E.van Wyk	#2217 A	South Africa, Western Cape, 3419CB, Stanford. Grootbos, 22 January 2007, *Anthony R. Magee, J.S.Boatwright, J.C.Manning & H.Lutzeyer 106* (NBG 0238439-0)	MW166358		
Capnophyllum macrocarpum Magee & B.-E.van Wyk	#1918 A	South Africa, De Hoop, *Anthony R. Magee 133* (ethanol-preserved collection at the Department of Botany and Plant Biotechnology, University of Johannesburg, Johannesburg, South Africa)			

Taxon	Accession ID	Voucher information	GenBank accession numbers		
			ITS	*rpoC1* intron	*rps16* intron
Cynorhiza typica Eckl. & Zeyh.	#2212 A	South Africa, Cape region, along road leading east of Steinkopf/Port Bolloth/Kosies road, 3200 ft, 29 March 1981, *D.Snijman 422* (NBG 122419)			
Dasispermum capense (Lam.) Magee & B.-E.van Wyk	#2215 A	South Africa, Tuinlaagte, Oorlogskloof, *Anthony R. Magee et al. 128* (JRAU)		MW166368	
Dasispermum capense (Lam.) Magee & B.-E.van Wyk	#2221 A	South Africa, Western Cape, 3219DC, 60 km NE of Ceres. Knolfontein. Swartruggens, 1208 m, 15 January 2009, *I.Jardine & C.Jardine 1080* (NBG 247796-0)			
Dasispermum hispidum (Thunb.) Magee & B.-E.van Wyk	#2220 A	South Africa, Western Cape, 3419AD, Stanford, Grootbos, near to main house, 22 January 2007, *Anthony R. Magee, J.S.Boatwright et al. 107* (NBG 0252209-0)			
Dasispermum hispidum (Thunb.) Magee & B.-E.van Wyk	#2236 A	Sandy Bay, 20 January 2001, *Anthony R. Magee & Boatwright 105* (ethanol-preserved collection at the Department of Botany and Plant Biotechnology, University of Johannesburg, Johannesburg, South Africa)			
Dasispermum perennans Magee & B.-E.van Wyk	#2222 A	South Africa, Cape, 3418AB, Hout Bay, above fisheries, 22 September 1984, *C.H.Stirton 10754* (NBG 130597-0)			
Dasispermum perennans Magee & B.-E.van Wyk	#2231 A	South Africa, Sandy Bay, Table Mountain National Park, *Anthony R. Magee & Boatwright 105* (JRAU, NBG). Specimen labelled as *Sonderina* sp.			
Dasispermum suffruticosum (P.J.Bergius) B.L.Burtt	#1919 A	South Africa, *34/06* (ethanol-preserved collection at the Department of Botany and Plant Biotechnology, University of Johannesburg, Johannesburg, South Africa)			
Dasispermum suffruticosum (P.J.Bergius) B.L.Burtt	#1920 A	South Africa, 8 September 1994 (ethanol-preserved collection at the Department of Botany and Plant Biotechnology, University of Johannesburg, Johannesburg, South Africa)			
Lefebvrea abyssinica A.Rich.	#2356 A	Tanzania, 7 miles from Babati on the Singida road, Mbulu District, Tanganyika, 5650 ft, 6 May 1962, *R.Polhill & S.Paulo 2366* (B 100844047)			
Nanobubon capillaceum (Thunb.) Magee	#2226 A	South Africa, Western Cape, 3419DB, on graded road leading to Boschoek house, 342 m, 16 January 2011, *N.J. van Berkel 1057* (NBG 265280-0)	MW166353	MW166366	MW166377
Nanobubon hypogaeum Magee	#2227 A	South Africa, Western Cape, 3423AA, 3-5 m down slope from fire break at corner of Villa Castrolini Wall, 175 m, 13 July 2010, *N.J. van Berkel 1014* (NBG 271684-0)	MW166354	MW166367	MW166378
Nanobubon strictum (Spreng.) Magee	#2228 A	South Africa, Western Cape, 3318DB, Paardeberg, between Wellington and Malmesbury, Vondeling, +- 1.8 km west of farm house next to 4x4 track, 608 m/772 m, 12 December 2012, *G.Nicolson & D.Roets 772* (NBG 276515-0)		MW166365	
Nanobubon strictum (Spreng.) Magee	#2235 A	9 October 1993, *B.-E. van Wyk 3500* (ethanol-preserved collection at the Department of Botany and Plant Biotechnology, University of Johannesburg, Johannesburg, South Africa)			
Notobubon capense (Eckl. & Zeyh.) Magee	#1925 A	South Africa, Betty's Bay, 21 December 2016, *A. Oskolski* & *K.Frankiewicz* (WA 0000049680) ex University of Johannesburg, Department of Botany and Plant Biotechnology AO136-16.			
Notobubon capense (Eckl. & Zeyh.) Magee	#2537 A	South Africa, Harold Porter Botanical Garden, Betty's Bay, *Anthony R. Magee et al. 37* (JRAU)			
Notobubon capense (Eckl. & Zeyh.) Magee	#2538 A	South Africa, Khamiesberg, Stalberg summit, *Anthony R. Magee et al. 43* (JRAU)			
Notobubon ferulaceum (Thunb.) Magee	#2540 A	South Africa, Plettenberg Bay, Perdekop, *P.J.D. Winter 158* (JRAU)			
Notobubon galbaniopse (H.Wolff) A.R.Magee	#2224 A	South Africa, Cape, Stellenbosch dist., Jakkalsvlei Jonkershoek List 3., 1300', 21 January 1964, *H.C.Taylor 5668* (NGB 0198315-0)			
Notobubon galbanum (L.) Magee	#2535 A	South Africa, Du Toit's Kloof Pass, *B.-E. van Wyk & P.J.D. Winter 3489* (JRAU)			
Notobubon gummiferum (L.) Magee	#2124 A	South Africa, Jan 1882 (WU 0095777)	MW166355		
Notobubon gummiferum (L.) Magee	#2536 A	South Africa, Duyvels and Voormansbosch, *Anthony R. Magee et al. 61* (JRAU)			

Taxon	Accession ID	Voucher information	GenBank accession numbers		
			ITS	*rpoC1* intron	*rps16* intron
Notobubon laevigatum (Aiton) Magee	#2539 A	South Africa, near Plettenberg Bay, *P.J.D. Winter 90* (JRAU)			
Notobubon pearsonii (Adamson) Magee	#2532 A	South Africa, 1 km north of Windpoort, Khamiesberg, *Anthony R. Magee 42* (JRAU)			
Notobubon pungens (E.Mey. ex Sond.) Magee	#2533 A	South Africa, 3 km south of Helderstroom prison on Brakfontein 123, *Anthony R. Magee et al. 50* (JRAU)			
Notobubon sonderi (M.Hiroe) Magee	#2225 A	South Africa, S. Cape, 3418BB (Simonstown), Jonkershoek Forest Reserve - south east slope of Triplets., ca 4600', 11 January 1970, *F.J.Kruger 1007* (NBG 202127-0)			
Notobubon striatum (Thunb.) Magee	#2534 A	South Africa, 300 m from entrance to Potberg, *Anthony R. Magee et al. 51* (JRAU)			
Notobubon tenuifolium (Thunb.) Magee	#2541 A	South Africa, Touwsberg, farm Wolwenfontein, *B.-E. van Wyk et al. 3430* (JRAU)			
Scaraboides manningii Magee & B.-E.van Wyk	#2218 A	South Africa, Western Cape, 3320AA, Tanqua National Park, E end of Elandsberg., 16 September 2006, *J.C.Manning 3061* (NBG 206897-0)	MW166356	MW166369	
Stenosemis angustifolia E.Mey. ex Harv. & Sond.	#2213 A	South Africa, 3128 BC. Hill above Mhlanfane Forest Station, N.W. of Umtata, 5000 ft, 31 January 1983, *O.M.Hilliard & B.L.Burtt 16324* (NBG 0143230-0)			
Stenosemis caffra (Eckl. & Zeyh.) Sond.	#2241 A	South Africa, Eastern Cape, 3325 BD, Zuurberg National Park, Ferniebrae, end of track at W entrance, 700 m, 8 January 1986, *B.-E. van Wyk & M. van Wyk 1210* (JRAU 868)			
**Tordyliinae**					
Heracleum sphondylium subsp. *spondylium* L.	#2059 A	Poland, Łódź Voivodeship, Łowicz County, Łowicz Commune, Niedźwiada village, at the road side between address Niedźwiada 70a and the water conditioning station, 15 July 2017, *Ł.Banasiak* (WA 0000050199)			
Pastinaca sativa L.	#2056 A	Poland, Łódź Voivodeship, Łowicz County, Łowicz Commune, about 50 meters SE from railway crossing of the road leading from Niedźwiada village to the trunk road no. 92 (former no 2) and a railway line from Łowicz to Kutno, at the roadside, 15 July 2017, *Ł.Banasiak* (WA 0000050196)			
Semenovia frigida (Boiss. & Hausskn.) Manden.	#2187 A	Iran, Tehran, Tuchal Mountains, 3730 m, 13 August 2007, *J.Noroozi 1305* (WU 0095808)			
Semenovia lasiocarpa (Boiss.) Manden.	#2184 A	Afghanistan, province Bamian, Band-i Amir, 2800-2900 m, 13 July 1962, *K.H.Rechinger & H.Riedl 18236* (WU 0095810)			
Tordylium maximum L.	#0716	Czech Republic, Moravia, Rabenstein by Znaim (Znojmo), 22 June 1909, *A.Oborny* (WA 0000051689)	MW166360	MW166372	MW166380
Trigonosciadium viscidulum Boiss. & Hausskn. ex Boiss	#2126 A	Iraq, Mountain Kuh-Sefin, near Erbil, higher parts, 16-1900 m, 21 May 1893, *J.Bornmüller 1274* (WU 0095750)	MW166359		

Taxon	GenBank accession numbers		
	ITS	*rpoC1* intron	*rps16* intron
**Sequences retrieved from GenBank**			
**Outgroup**			
Echinophora chrysantha Freyn & Sint.	AF077883		
***Cymbocarpum* clade**			

Taxon	GenBank accession numbers		
	ITS	*rpoC1* intron	*rps16* intron
Cymbocarpum anethoides DC. Ex C.A.Mey.	EU169253		
Cymbocarpum alinihatii Menemen & Cingay	KY989959		
Cymbocarpum marginatum Boiss.	EU169293		
Cymbocarpum wiedemannii Boiss.	GU291352		
Ducrosia assadii Alava	DQ427043		
Ducrosia flabellifolia Boiss.	DQ427051		
***Lefebvrea* clade**			
Afroligusticum aculeolatum (Engl.) P.J.D.Winter	KJ173906		
Afroligusticum elgonense (H.Wolff) P.J.D.Winter	KJ173907		
Afroligusticum elliotii (Engl.) C.Norman	DQ516377		
Afroligusticum petitianum (A. Rich.) P.J.D.Winter	AM408869		
Afroligusticum piovanii (Chiov.) Kljuykov & Zakharova	KF164290		
Afrosciadium magalismontanum (Sond.) P.J.D.Winter	AM408876		
Capnophyllum leiocarpon (Sond.) J.C.Manning & Goldblatt	FM201526		FM201545
Capnophyllum lutzeyeri Magee & B.-E.van Wyk			FM201543
Capnophyllum macrocarpum Magee & B.-E.van Wyk	FM201529		FM201547
Cynorhiza typica Eckl. & Zeyh.	AM408865		FM201556
Dasispermum capense (Lam.) Magee & B.-E.van Wyk	FM201516		FM201532
Dasispermum hispidum (Thunb.) Magee & B.-E.van Wyk	FM201521		FM201536
Dasispermum humile (Meisn.) Magee & B.-E.van Wyk	AM408873		FM201538
Dasispermum suffruticosum (P.J.Bergius) B.L.Burtt	AM408872		AY838417
Dasispermum tenue (Sond.) Magee & B.-E.van Wyk	FM201517		FM201539
Lefebvrea abyssinica A.Rich.	DQ516376		
Lefebvrea grantii (Kingston ex Oliv.) S. Droop	AM408881		

Taxon	GenBank accession numbers		
	ITS	*rpoC1* intron	*rps16* intron
Lefebvrea longipedicellata Engl.	AM408877		
Nanobubon strictum (Spreng.) Magee	AM408862		AM408862
Notobubon capense (Eckl. & Zeyh.) Magee	AM408850		FM201555
Notobubon ferulaceum (Thunb.) Magee	AM408858		AY838434
Notobubon galbaniopse (H.Wolff) A.R.Magee	AM408853		
Notobubon galbanum (L.) Magee	AM408844		
Notobubon gummiferum (L.) Magee			FM201554
Notobubon laevigatum (Aiton) Magee	AM408860		
Notobubon pearsonii (Adamson) Magee	AM408867		AY838436
Notobubon pungens (E.Mey. ex Sond.) Magee	AM408856		AY838437
Notobubon striatum (Thunb.) Magee	AM408855		
Notobubon tenuifolium (Thunb.) Magee	AM408848		FM201553
Stenosemis angustifolia E.Mey. ex Harv. & Sond.	AM408875		
Stenosemis caffra (Eckl. & Zeyh.) Sond.	AM408874		AY838444
**Tordyliinae**			
Heracleum sphondyllium L.	MK050079		AF164800
Pastinaca sativa L.	EU185668	U36299	AF110538
Semenovia frigida (Boiss. & Hausskn.) Manden.	DQ427039		
Semenovia lasiocarpa (Boiss.) Manden.	FJ861182		
Zosima orientalis Hoffm.	AF008628		AF164806

1. Thiers BM. (continously updated) Index Herbariorum: A global directory of public herbaria and associated staff. New York Botanical Garden’s Virtual Herbarium. http://sweetgum.nybg.org/ih/. 2013.
