# Supplementary material for: Derived woodiness and annual habit evolved in African umbellifers as alternative solutions for coping with drought
Source: BMC Plant Biol. 2021 Aug 20;21:383. doi: 10.1186/s12870-021-03151-x (PMC8377965; doi:10.1186/s12870-021-03151-x)
Supplement: Supplementary file 2 — Additional file 2. Maximum likelihood phylogenetic trees with bootstrap values. [file 12870_2021_3151_MOESM2_ESM.pdf]

Frankiewicz *et al.* 2021 – Additional file A2

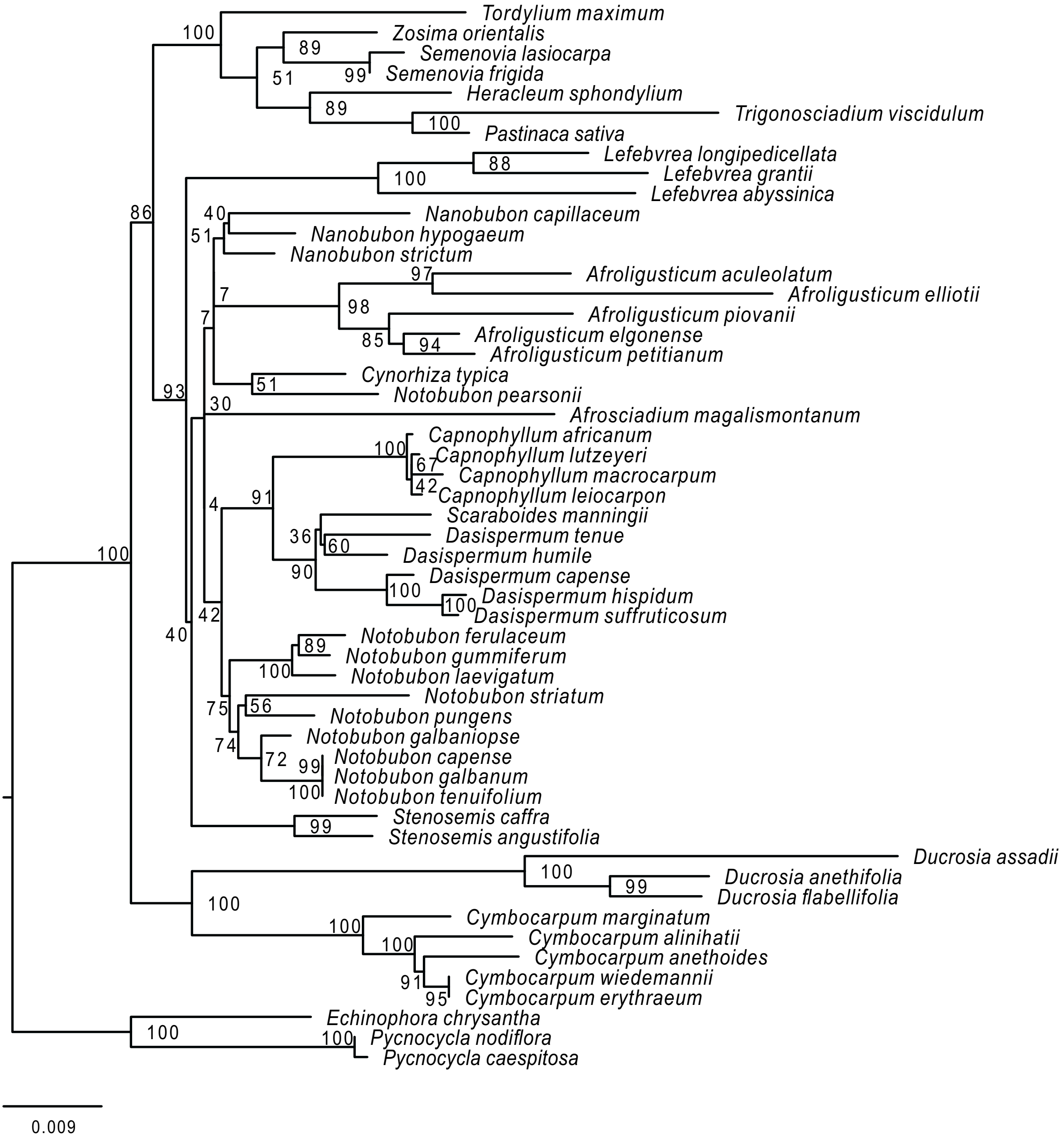

Additional file A2: Maximum likelihood phylogenetic tree inferred from combined sequences of nrDNA ITS, and plastid DNA *rpoC1* and *rps16* introns. Bootstrap support is given along the branches.
