# Supplementary material for: Derived woodiness and annual habit evolved in African umbellifers as alternative solutions for coping with drought
Source: BMC Plant Biol. 2021 Aug 20;21:383. doi: 10.1186/s12870-021-03151-x (PMC8377965; doi:10.1186/s12870-021-03151-x)
Supplement: Supplementary file 3 — Additional file 3. Detailed wood descriptions. [file 12870_2021_3151_MOESM3_ESM.pdf]

Additional file A3 – Wood descriptions of the *Lefebvrea* clade genera and outgroup studied by Frankiewicz *et al.* 2021: Derived woodiness and annual habit evolved in African umbellifers as alternative solutions for coping with drought.

For figures and figures legends see the original publication.

Details of wood anatomy of the *Lefebvrea* clade representatives

Afrosciadium — wood anatomical descriptions are based on *A. magalismontanum* and *A. platycarpum*.

Growth ring boundaries are absent. Wood is diffuse-porous, vessels are circular to oval, angular, and confined mostly to fascicular regions. They are narrow 19–26(–45) µm in tangential diameter and their number is markedly higher in *A. magalismontanum* (275 per mm^2), than in *A. platycarpum* (71 per mm^2). Vessels are disposed without any distinct pattern: partly solitary (11%–31%) and in small clusters of 3–4.3(–17) vessels. Vessel walls are thicker in *A. magalismontanum* (3.0–)4.9(–8.7) µm than in *A. platycarpum* (1.7–)3.3(–5.5) µm. Vessel element length was measured only in *A. platycarpum*: (214–)392(–721) µm. Perforation plates are exclusively simple. Intervessel pitting is clearly scalariform in *A. magalismontanum* with pits (1.3–)2.3(–3.9) µm wide in vertical diameter, and mostly scalariform in *A. platycarpum* with pits (2.6–)3.7(–5.1) µm. Vessel-axial parenchyma pitting is similar to intervessel pitting and clearly bordered in *A. platycarpum*. In *A. magalismontanum* no axial parenchyma associated with vessels was observed.

The background tissue consists of libriform, non-septate, very thin-walled fibres that are (279–)475(–1536) µm long, and with walls (1.8–)2.5(–3.8) µm thick in *A. platycarpum*. In *A. magalismontanum* the background tissue consists of pervasive parenchyma with starch grains

in some cells: no clear strands of cells can be recognised and no axial parenchyma or rays can be distinguished from the pervasive, background tissue.

Axial parenchyma in *A. platycarpum* in scanty paratracheal in strands of 1–2 cells, to vasicentric with sheaths usually 1 cell thick. Rays are rare, uni- and narrow multiseriate 3.4(–6) cells wide. Multiseriate rays height varies considerably (170–)960(–2000) µm. They are composed of upright cells.

Capnophyllum — wood anatomical descriptions are based on *C. africanum, C. macrocarpum, C. leiocarpon* (only radial section was available), and *C. lutzeyeri*.

Growth ring boundaries are absent (Fig. 3c, 4a). Wood is diffuse-porous, but in *C. lutzeyeri* secondary xylem is too narrow to confirm it. Vessels are circular to oval, and slightly angular. They are narrow 16–23(–34) µm in tangential diameter and numerous to very numerous (mean number of vessels per mm^2 ranges from 240 in *C. africanum* to 424 in *C. macrocarpum*; in *C. lutzeyeri* the secondary growth is so narrow that measurement may be unreliable). Vessel are disposed in vague to distinct radial pattern, partly solitary (18–27%) or in radial multiples of ca. 3.3(–10) vessels. Vessel walls are (1.3–)2.1–3.0(–5.0) µm thick. Vessel elements are (133–)281–303(–546) µm long. Perforation plates are exclusively simple. Intervessel pitting is alternate in *C. africanum* with pits (2.7–)3.7(–4.7) µm wide in vertical diameter, in the remaining species intervessel pitting is alternate (or transitional between opposite and alternate) with pits (2.2–)3.0–4.0(–6.6) µm wide in vertical diameter. Vessel-axial parenchyma pitting is similar to intervessel pitting and have pits clearly bordered in *C. africanum* and *C. lutzeyeri*; in *C. macrocarpum* and *C. leiocarpon* it was not observed.

The background tissue is fibrous and is composed of libriform, non-septate, thin- to thick-walled fibres ca. (285–)412–509(–698) µm long, with walls (1.3–)2.1–2.2(–4.3) µm thick (Fig. 3c, 4a). Axial parenchyma is very scanty paratracheal, hardly distinguishable from the

background tissue in *C. africanum*, scanty paratracheal in strands of ca. 3–5 cells in *C. macrocarpum* and *C. lutzeyeri*. Axial parenchyma was not observed in *C. leiocarpon*.

Rays were not observed in *C. macrocarpum*. In *C. africanum* files of wider cells, similar to fibres were observed in transverse sections. In *C. lutzeyeri* rays are rare and uni- to triseriate. Ray cellular composition was observed only in *C. leiocarpon* – they consist of upright, square, and procumbent cells mixed throughout the ray body.

Cynorhiza — wood anatomical description is based on a sample of *C. typica*.

Growth ring boundaries are absent. Wood is diffuse-porous, vessels are circular to oval, and slightly angular (Fig. 4d). They are ca. 47(–70) µm wide, and numerous (ca. 180 vessels per mm^2). Vessels are disposed more or less homogenously without any distinct pattern. Groups of vessels are separated by wide, parenchymatous rays being prolongations of medullary rays. Vessel walls are (3.8–)6.7(–10.3) µm thick. Vessel elements are (110–)205(–296) µm long. Perforation plates are exclusively simple. Intervessel pitting is mostly scalariform (sometimes transitional between opposite and alternate), with pits (1.9–)3.8(–6.3) µm in vertical diameter. Vessel-ray or vessel-axial parenchyma pitting were not observed.

The background tissue consists of pervasive parenchyma, highly distorted in the available sample. In the selected sections, a narrow layer of very thin-walled fibres forming a cap over vessels can be observed. No axial parenchyma can be distinguished. Rays are uni- and narrow multiseriate (≤ 5 cells wide).

Dasispermum — wood anatomical descriptions are based on *Dasispermum capense*, *Dasispermum hispidum*, *Dasispermum perennans,* and *Dasispermum suffruticosum* (Fig. 4e-f). Additionally, a root sample of *D. perennans* (#2231) was examined.

Growth ring boundaries are absent (*D. capense, D. hispidum, D. perennans*) to present and indistinct to distinct (*D. perennans* root #2231, *D. suffruticosum*): they are marked with layers of narrower fibres, well-stained with safranin (*Dasispermum perennans* root #2231) or a band of marginal parenchyma (4 cell layers thick; in *D. suffruticosum*). Wood is diffuse-porous. Vessels are circular to oval and slightly angular. Vessels are narrow, 10–21(–32) µm and numerous to very numerous (from 137 vessels per mm^2 in *D. capense* #2215 to 439 in *D. perennans*; in *D. suffruticosum* and the other sample of *D. capense* #2221 the vessel frequency is higher and reaches almost 850 vessels per mm^2, but this is a result of large groups of vessels present in the juvenile wood). Vessel grouping is indistinct – partly solitary (8%–30%) and in small clusters and radial multiples of 5(–15) vessels. The secondary xylem was too narrow to determine vessel arrangement in one sample of *D. capense* (#2215) and *D. hispidum* (#2236). In the remaining samples (including the root of #2231), a (vague) radial pattern was observed – most likely a juvenile trait. In *D. suffruticosum* (#1919) vessels in the inner wood are disposed without any distinct pattern, and a (vague) radial pattern, similar to the one observed in other species, is established only in the outer wood. Vessel walls are (1.4–)2.1–4.0(–6.2) µm thick, and vessel elements are (76–)208–279(–743) µm long. Perforation plates are exclusively simple. Intervessel pitting is (mostly) alternate (*D. hispidum, D. perennans, D. perennans* root #2231, *D. suffruticosum*; Fig. 4f), or transitional with some scalariform (*D. capense* #2215) and opposite (*D. capense* #2221) pitting. Pits are (1.4–)2.6–3.9(–5.6) µm in vertical diameter. In most cases they have slit-like apertures (lens-like apertures in *D. capense*). Vessel-axial parenchyma pitting is clearly bordered and similar to intervessel pitting.

The background tissue is of two types: (1) In *D. capense*, *D. hispidum*, and *D. perennans,* including root sample #2231, only fibrous background in secondary xylem was observed. Additionally, a well-seen parenchymatous metaxylem was present in *D. perennans* stem sample. (2) In *D. suffruticosum* innermost area of secondary xylem is parenchymatous,

while the remaining (dominant) part is fibrous. Fibres are thin- (*D. capense*) or thin- to thick-walled (*D. hispidum*: thinner walled in: #2220, thicker-walled in #2236; *D. perennans,* including root sample #2231, and *D. suffruticosum*) with wall thickness of (1.1–)1.9–2.9(–4.7) µm. They are (116–)346–424(–607) µm long.

Axial parenchyma is scanty paratracheal to very scanty paratracheal in short, rarely observed strands (3 cells long in *D. hispidum*, 5 cells in *D. perennans*, 2–5 in *D. perennans* root #2231). In *Dasispermum suffruticosum* axial parenchyma was not observed.

In *D. capense* files of cells wider than surrounding fibres were observed in one sample (#2215) – a condition close to raylessness. In the remaining samples uni- and rare (≤ 5 per mm) narrow multiseriate (≤ 8 cells wide) rays were observed. More numerous (9.1 per mm) multiseriate rays (≤ 6 cells wide) were observed only in the root sample (*D. perennans* #2231). Multiseriate rays are (102–)418–510(–1400) µm high. In the root sample rays commonly exceeded the height of the sections. Rays cellular composition was observed clearly only in the root sample (procumbent and square cells intermixed with some upright cells throughout the ray body). In the remaining species they are most likely composed of square and upright cells.

Nanobubon — wood anatomical descriptions are based on *N. capillaceum* and *N. strictum*, and a root sample of *N. hypogeum* (#2227).

Growth ring boundaries are absent. Wood is diffuse-porous, vessels are circular to oval, and angular. Vessels are narrow: 22–35 µm, and few (45–68 per mm^2). Only in the root sample of *N. hypogeum* vessels are very numerous (ca. 450 per mm^2). Vessel grouping is indistinct: partly solitary (3–8%), and in small clusters and radial multiples ca. 5(–9) vessels. Only in a sample of *N. strictum* (#2228) bigger vessel clusters were observed (up to 27 vessels). Vessels in the root sample (*N. hypogeum* #2227) are disposed more or less homogenously; in one sample of *N. strictum* (#2235) a vague dendritic pattern was observed, and in the remaining samples

the pattern is indistinct. Vessel wall thickness is (2.4–)5.2–6.0(–0.5) µm, and vessel elements are (106–)293–592(–850) µm long. Perforation plates are exclusively simple. Intervessel pitting could not be clearly observed in *N. capillaceum*. In *N. strictum* it is alternate with pits (1.1–)2.6–5.2(–7.4) µm wide in vertical diameter. In the root sample of *N. hypogeum* it is mostly scalariform (occasionally also transitional and opposite), with pits (2.1–)3.4(–4.7) µm wide.

The background tissue in *N. capillaceum* and *N. strictum* is fibrous: it consists of thick- to very thick-walled fibres, (200–)459–758(–1030) µm long. Fibre walls are (3.0–)4.7–6.8(–0.3) µm thick. In *N. strictum* axial parenchyma is locally pervasive. In root sample of *N. hypogeum* the background tissue consists exclusively of pervasive parenchyma.

Axial parenchyma is scanty paratracheal to almost vasicentric (*N. capillaceum*, *N. strictum* #2228), and mostly vasicentric (*N. strictum* #2235). In both cases in short strands (precise length was difficult to establish in dried material). In *N. hypogeum* axial parenchyma could not be distinguished from the pervasive background parenchyma.

Rays were not observed in the root sample (*N. hypogeum*). In *N. capillaceum* rays were very distorted and observed only in transverse section, but they were similar to better-preserved rays in *N. strictum*, where they are scarce, uni- and narrow multiseriate (≤ 4), and (223–)520–635(–1376) µm tall. Rays are composed of procumbent and square cells, with some upright cells intermixed throughout the ray body.

Notobubon — wood descriptions are based on: *N. capense*, *N. ferulaceum*, *N. galbaniopse*, *N. galbanum*, *N. gummiferum*, *N. laevigatum*, *N. pearsonii*, *N. pungens*, *N. sonderi* (a very young shoot), *N. striatum*, *N. tenuifolium*.

Growth ring boundaries are absent (*N. capense* #1925, *N. galbanum*, *N. gummiferum*, *N. sonderi*), present and indistinct (*N. ferulaceum*, *N. galbaniopse*, *N. laevigatum*, *N. pungens*, *N. striatum*, *N. tenuifolium*; Fig. 5a), to present and distinct (*N. capense* #2537, #2538, *N.*

pearsonii; Fig. 5b). Boundaries are marked by differences in vessel size (*N. capense* #2537, #2538, *N. laevigatum*, *N. pearsonii*, *N. pungens*, *N. striatum* #2534, *N. tenuifolium*; Fig. 5b), radially flattened fibres (*N. ferulaceum*, *N. tenuifolium*; Fig. 5a), change in fibre wall thickness from thin- to thick-walled between earlywood and latewood (*N. capense*, *N. ferulaceum*, *N. pearsonii*, *N. pungens*, *N. tenuifolium*; Fig. 5b), a band of marginal parenchyma (*N. laevigatum*, *N. pungens*), or an occasional uniseriate layer of marginal parenchyma (*N. striatum*). Wood is diffuse-porous (most species; Fig. 5a-b) to semi-ring porous (*N. pearsonii*; Fig. 3d). Vessels are rounded, sometimes angular in outline, rather narrow (tangential diameter: 14–75 µm). Vessel frequency ranges from 40 per mm^2 in *N. capense* to 371 per mm2 in *N. striatum*, to 549 per mm^2 in *N. gummiferum* #2124. Vessels are grouped in radial multiples or clusters of 2–30 (up to 45 in *N. striatum*) together with a low percentage of solitary vessels, more than 10% of solitary vessels is found only in *N. capense*, *N. galbaniopse*, *N. galbanum*, *N. gummiferum* #2536, *N. sonderi*, and *N. tenuifolium*. Vessel arrangement is indistinct (*N. capense* #1925; *N. galbaniopse*), tending to radial (*N. capense* #2537, #2538, *N. galbanum*, *N. gummiferum*, *N. tenuifolium*; Fig. 5b), radial (*N. ferulaceum*, *N. gummiferum* #2124, *N. laevigatum*, *N. pungens*, *N. sonderi*) or diagonal to dendritic (*N. pearsonii*, *N. striatum*; Fig. 3d). In *N. capense*, *N. ferulaceum*, *N. gummiferum*, *N. galbaniopse*, *N. galbanum*, *N. laevigatum*, *N. pungens*, *N. sonderi*, and *N. striatum* vessels are localised in fascicular zones, while interfascicular regions are composed (almost) exclusively of fibres. Vessel walls are 2–5 µm thick (≤ 7 µm in *N. capense* and *N. laevigatum*). Vessel elements are of similar length in all species: (77–)189–347(–606) µm. Perforation plates are exclusively simple (an abhorrent reticulate perforation plate was observed in *N. tenuifolium*; Fig. 5f). Intervessel pitting is (mostly or exclusively) alternate (Fig. 5d), pits are small, 4.5–6.5(–8.3) in vertical diameter (smaller in *N. striatum*, 3.5–4.0), with rounded, sometimes oval margins and slit-like apertures. In *N. sonderi* intervessel pitting is transitional between alternate and opposite to scalariform. Vessel-ray and

vessel-axial parenchyma pitting is usually similar to intervessel pitting (Fig. 5f), pits are almost exclusively narrow-bordered, occasionally with indistinct borders in *N. pearsonii*. Helical thickenings are present (*N. ferulaceum*, *N. laevigatum*, *N. pearsonii*, *N. pungens*, *N. striatum,*) or absent (*N. capense*, *N. galbaniopse*, *N. galbanum*, *N. gummiferum*, *N. sonderi*, *N. tenuifolium*).

The background tissue consists of libriform fibres (Fig. 3d, 4a–c). They are moderately thick-walled throughout (*N. capense, N. galbanum*, *N. gummiferum*, *N. laevigatum*, *N. pungens*, *N. sonderi*, *N. tenuifolium*), or moderately thick-walled becoming very thick-walled in latewood (*N. ferulaceum*, *N. pearsonii*, *N. striatum*; Fig. 5c), fibre walls are (1.3–)2–7(–10.1) µm thick. Fibres are non-septate in all species. In *N. sonderi* fibres are poorly lignified.

Axial parenchyma is scanty paratracheal, in incomplete sheaths only (*N. capense* #2537, #2538, *N. ferulaceum*, *N. galbaniopse*, *N. pearsonii*, *N. pungens*, *N. sonderi*, *N. striatum*; Fig. 5c), or in incomplete to sometimes complete sheaths (*N. capense* #1925, *N. galbanum*, *N. gummiferum*, *N. laevigatum*, *N. tenuifolium*). Marginal parenchyma is present as an occasional uniseriate layer (*N. striatum*), or in bands of 2–3 cells wide (*N. laevigatum, N. pungens*). Axial parenchyma cells are fusiform or in strands of 2–4(–6) cells (Fig. 5d).

Rays are 4–7 per mm, uni- and multiseriate of 2–3 cells in width (up to 4 in *N. capense* #1925, *N. galbaniopse*, and *N. tenuifolium*; Fig. 5d). Ray height exceeds 1 mm only in *N. capense* #2537, #2538, *N. ferulaceum*, *N. gummiferum*, *N. pungens*, *N. tenuifolium* and, those of *N. pearsonii* never exceed more than 0.3 mm in height. Rays are composed mostly of square and upright cells with some procumbent cells mixed throughout (*N. capense* #2537, #2538, *N. ferulaceum*, *N. galbaniopse*, *N. galbanum*, *N. gummiferum*, *N. laevigatum*, *N. pungens*, *N. sonderi*, *N. striatum*, and *N. tenuifolium*; Fig. 5e), or the body is mainly of procumbent cells with 1–4 square and rarely upright cells in the margins (*N. capense* #1925), or composed mostly of procumbent cells with some square cells in the body and occasionally upright cells in the

marginal rows (*N. pearsonii*). Silica bodies were found in ray cells of *N. ferulaceum, N. gummiferum, N. laevigatum, N. pungens, N. striatum,* and *N. tenuifolium*.

Scaraboides — wood description is based on a root sample of *S. manningii* (Fig. 4c).

Growth ring boundaries are absent. Wood is diffuse-porous. Vessels are angular, very narrow 14(–23) µm, and numerous (ca. 258 per mm^2). Vessel disposition is indistinct, partly in radial multiples or small clusters of 3(–10) vessels, partly solitary (17%). Vessel wall is (1.5–)2.6(–4.3) µm thick. Vessel elements are (98–)233(–336) µm long. Perforation plates are exclusively simple. Intervessel pitting is opposite to alternate, occasionally scalariform with lens-like apertures. Pits are (1.6–)2.9(–4.8) µm in vertical diameter. Vessel-axial parenchyma pitting is similar to intervessel pitting and clearly bordered. Vessel-ray pitting was not observed.

The background tissue consists of thin-walled, non-septate fibres, (158–)374(–1418) µm long. Fibre walls are (1.4–)2.1(–2.9) µm thick. Axial parenchyma is scanty paratracheal in strands of 5–6 cells. Wood is almost rayless, very few uniseriate rays were found – they are composed of upright and square cells.

Stenosemis — wood descriptions are based on *S. angustifolia* and *S. caffra*.

Growth ring boundaries are absent in *S. caffra*, and present and indistinct in *S. angustifolia*: marked by fibres of wider lumen. Wood is diffuse-porous. Vessels are slightly angular, very narrow (11–12 µm in mean tangential diameter), and very numerous (in *S. caffra* 341 per mm^2, in *S. angustifolia* 597 per mm^2). Vessels disposition is indistinct in *S. angustifolia*, tending to radial in *S. caffra*. In both species vessels are partly solitary (7%–21%), partly in radial multiples and small clusters of 4–7(–20) vessels. Vessel wall is (1.5–)2.6–3.7(–6.5) thick. Vessel elements are of similar length: (120–)259(–387) µm. Perforation plates are exclusively simple. Intervessel pitting is alternate to opposite, with slit-like apertures and pits (1.0–)2.1–

2.8(–3.9) µm wide in vertical diameter. Vessel-axial parenchyma and vessel-ray pitting is similar to intervessel pitting (in *S. caffra* mostly alternate) and clearly bordered. Very fine helical thickenings or sculpturing of vessel walls was observed in both species.

The background tissue consists of non-septate fibres. In *S. angustifolia*, they are very thick-walled near the pith, and thin- to thick-walled towards the outside of the stem. In *S. caffra* all fibres are thick-walled. Fibres are of similar length in both species: (196–)314–373(–553) µm, and their walls are (1.5–)2.9–4.3(–7.3) µm thick. Additionally, in *S. caffra* a very narrow cylinder of parenchymatous wood is present near the pith.

Axial parenchyma is scanty paratracheal. Length of strands could not be determined. Studied sample of *S. caffra* is rayless, while in *S. angustifolia* rays are uni- and narrow multiseriate 3.5(–6) cells wide. There are ≤ 5 multiseriate rays per mm, and their height ranges (221–)931(–1884) µm. Rays are composed of upright and square cells.

Wood anatomy of the remaining Tordylieae clades

Tordyliinae — wood descriptions are based on *Heracleum sphondylium*, *Pastinaca sativa* (Fig. 6b-d), *Trigonosciadium viscidulum*, *Semenovia lasiocarpa* (Fig. 4b). In all samples except for *H. sphondylium*, a complete cylinder of secondary growth is present. In *H. sphondylium*, the secondary growth is only initiated in the fascicular regions (it consists mostly of fibres with few secondary vessels), while the interfascicular regions are still occupied by medullary rays (Fig. 3a). Nevertheless, the cambial activity in this species is evident. *Heracleum sphondylium* was excluded from the following description.

Growth ring boundaries are absent. Wood is diffuse-porous (Fig. 6b). Vessels are mostly oval in *P. sativa*, mostly circular in *S. lasiocarpa*, and circular to oval in *T. viscidulum*. They are narrow (mostly 15–29 µm) and numerous (110–160 per mm^2 in *P. sativa* and *T. viscidulum*, to very numerous in *S. lasiocarpa* 710 per mm^2). Vessels are grouped in radial multiples and

small clusters of 3–5(–17) in *P. sativa* and *T. viscidulum*. In *S. lasiocarpa* vessels are disposed homogenously throughout the secondary growth. Vessel arrangement is indistinct in *S. lasiocarpa* and *T. viscidulum* and vague radial in *P. sativa*. Vessel walls are (1.6–)2.7–2.9(–5.0) µm thick. Vessel element length spans (69–)178–275(–437) µm. Perforation plates are exclusively simple. Intervessel pitting is alternate in *P. sativa* and *T. viscidulum* with pits (2.9–)4.0–4.2(–5.6) µm wide in vertical diameter, and scalariform in *S. lasiocarpa* with pits (2.6–)4.3(–5.9) µm. Vessel-axial parenchyma and vessel-ray pitting were observed only in *P. sativa* and they are similar to intervessel pitting, and clearly bordered.

The background tissue consists of thin- to thick-walled fibres in *P. sativa* and *T. viscidulum* with fibres (166–)260(–320) µm long and with walls (1.2–)1.8(–2.5) µm in *T. viscidulum* and (3.0–)4.2(–5.9) µm thick in *P. sativa*. In *S. lasiocarpa* the background tissue consists of pervasive parenchyma with irregular patches of thick- to very thick-walled fibres (almost lacking lumen; Fig. 4b). Its fibres are (240–)439(–1101) µm long, and their walls are (3.5–)5.1(–7.9) µm thick.

Axial parenchyma is scanty paratracheal in strands of (2–)4–5 in *P. sativa*, in *T. viscidulum* the length of strands could not be determined (Fig. 6b-d). In *S. lasiocarpa* axial parenchyma could not be distinguished from the background tissue (Fig. 4b).

Rays are uni- and narrow multiseriate 3.4(–8) cells wide in *P. sativa* (Fig. 6c–d), multiseriate in *T. viscidulum* (ca. 2–5 cells, specimen was too distorted to count precisely). In *S. lasiocarpa* rays could not be recognised in transverse sections, but uniseriate rays were occasionally observed in longitudinal sections.

Cymbocarpum clade — wood descriptions are based on *Ducrosia anethifolia* (Fig. 6e) and a transverse section of *D. flabellifolia* (Fig. 3e).

Growth ring boundaries are absent. Wood is diffuse-porous. Vessels are mostly circular and rounded. They are narrow (ca. 20–24 µm) and very numerous (473–575 per mm^2). Vessel are grouped in small clusters and clear radial multiples in *D. anethifolia*, and in *D. flabellifolia* they do not form any distinct groupings. Vessel arrangement is indistinct closer to the pith, and vague radial in outer parts of *D. anethifolia*. In *D. flabellifolia* vessels are disposed more or less homogenously throughout the secondary growth (similarly to *S. lasiocarpa*). Vessel walls are (2.5–)3.5–4.8(–6.9) µm thick (thinner in *D. flabellifolia*, thicker in the latter species). Due to the scarcity of material vessel elements length was measured only in *D. anethifolia*, they are (77–)262(–388) µm long. Traits visible in longitudinal sections were observed only in *D. anethifolia*: perforation plates are exclusively simple. Intervessel pitting is alternate, rarely transitional. Pits are (3.8–)5.1(–6.3) µm wide in vertical diameter. Vessel-axial parenchyma and vessel-ray pitting is similar to intervessel pitting.

The background tissue in *D. anethifolia* consists of thin-walled fibres (250–)386(–644) µm long and with walls (1.6–)2.5(–3.6) µm thick. In *D. flabellifolia* the background tissue consists most likely of pervasive parenchyma.

Axial parenchyma in *D. flabellifolia* could not be distinguished from the background tissue. In *D. anethifolia* it is scanty paratracheal in strands (length of strands could not be precisely determined). Rays could not be distinguished from the background tissue in *D. flabellifolia*. In *D. anethifolia* rays are uni- and bi- to triseriate. They are composed of upright and square cells.

Wood anatomy of the outgroup

Wood descriptions of the outgroup are based on three species of *Pycnocycla* (Echinophoreae): *P. aucheriana*, *P. caespitosa*, and *P. nodiflora* (Fig. 3f, 6f)

Growth ring boundaries are present and distinct, marked by (1–)2–3 layers of, locally discontinuous, marginal parenchyma (Fig. 3f); in *P. caespitosa* also by 2–4 layers of radially flattened fibres, and in *P. nodiflora* locally also by a change between very thick-walled fibres in latewood and thick-walled fibres in earlywood. Wood is diffuse-porous. Vessels are circular to oval and rounded. They are narrow (21–33 µm) and numerous (115–229 vessels per mm^2). Vessel groupings are indistinct: partly solitary (18%–33%), partly in small clusters of 3–5(–11) vessels. Vessel arrangement is indistinct to vague dendritic (best seen in *P. nodiflora*). Vessel walls are (2.0–)3.5–4.5(–6.0) µm thick. Vessel elements lengths are very similar among species (87–)193–195(–384) µm. Perforation plates are exclusively simple. Intervessel pitting is alternate, pits are (2.3–)3.8–4.5(–5.8) µm wide in vertical diameter. Vessel-axial parenchyma and vessel-ray pitting are similar to intervessel pitting.

The background tissue consists of thin- to thick-walled, locally also very thick-walled (especially in *P. nodiflora*; Fig. 3f), fibres. They are (116–)326–364(–1185) µm long, and their walls are (2.1–)3.3–3.6(–5.5) µm thick.

Axial parenchyma is scanty paratracheal to vasicentric (especially in *P. nodiflora*; Fig. 3f) in strands of 2–5 cells. Rays are uni- and narrow multiseriate 3.9(–7) cells wide (Fig. 6c–d). Their height is similar among species (207–)478–486(–1262) µm tall. Rays are composed of procumbent and some square and upright cells intermixed throughout the ray body.
