# Supplementary material for: Derived woodiness and annual habit evolved in African umbellifers as alternative solutions for coping with drought
Source: BMC Plant Biol. 2021 Aug 20;21:383. doi: 10.1186/s12870-021-03151-x (PMC8377965; doi:10.1186/s12870-021-03151-x)

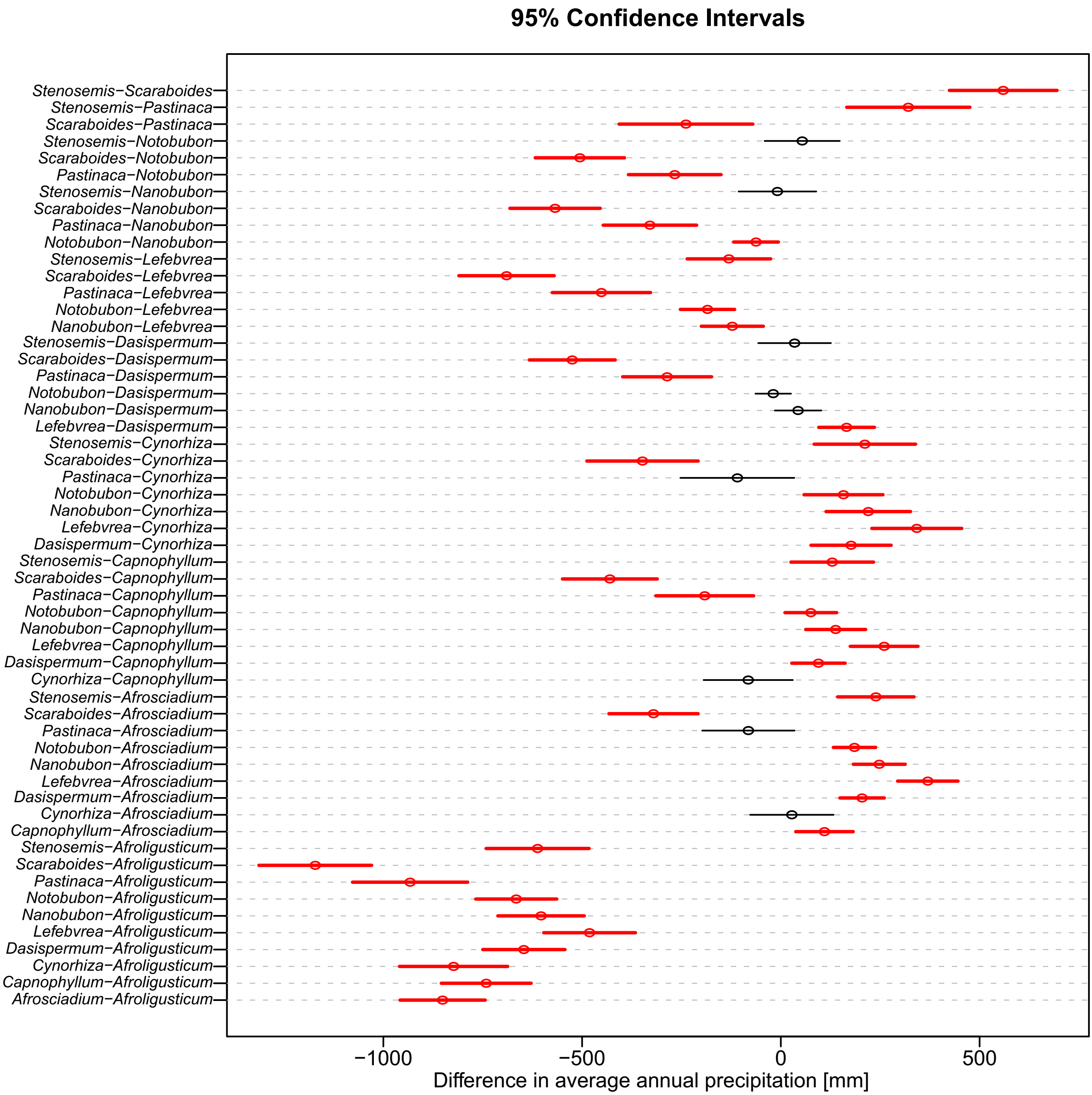

Additional file A4: A comparison of 95% confidence intervals for difference in means of the first (PC1) and the second (PC2) primary components backprojected onto annual precipitation and mean annual temperature dimensions of the bioclimatic space between pairs of genera within *Lefebvrea* clade and also *Pastinaca* outgroup. The differences that are exclusively positive or negative (i.e., excluding 0) are marked in red.

Frankiewicz *et al.* 2021 – Additional file A4

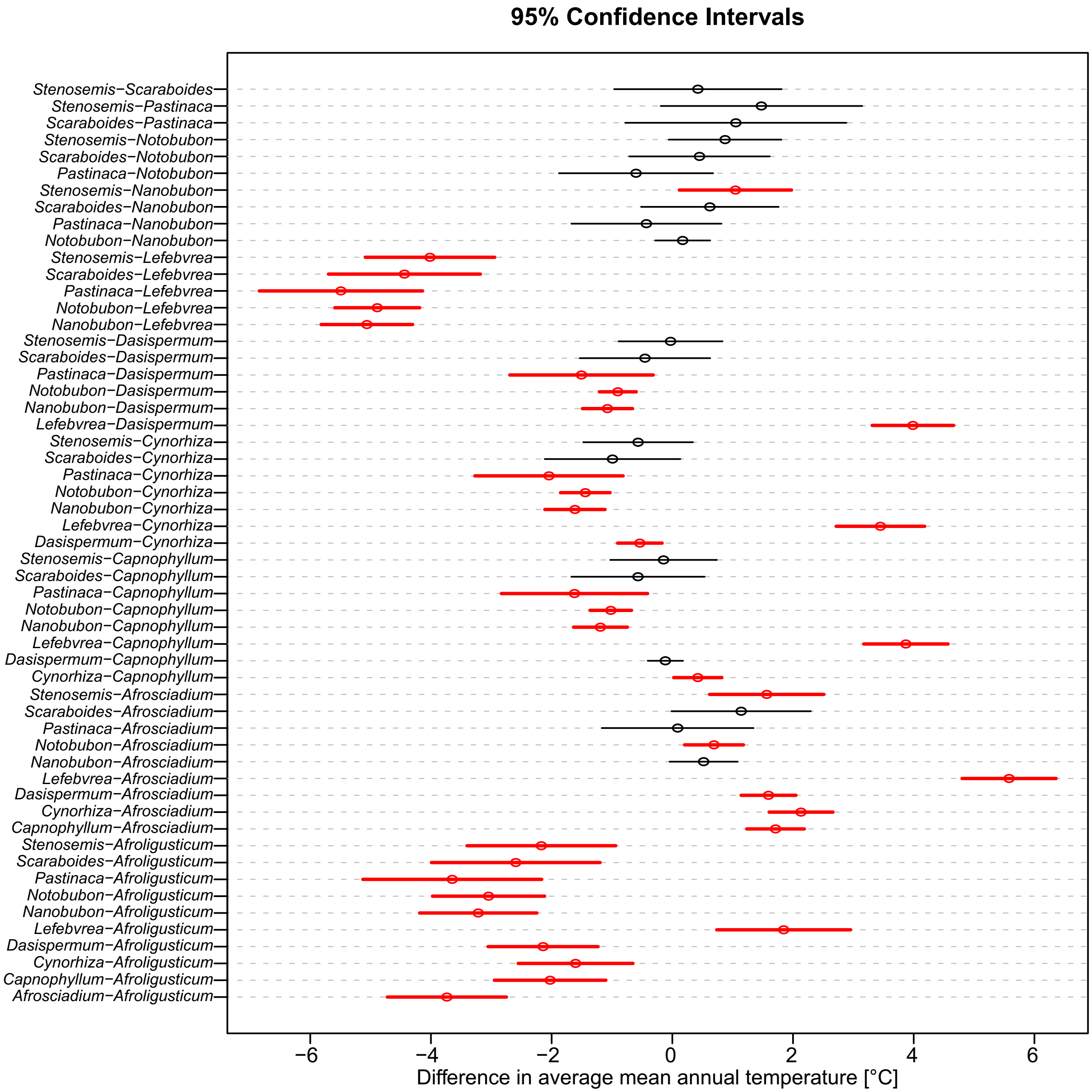

Supplement: Supplementary file 4 — Additional file 4. Plots of 95% confidence intervals for the difference of means in the first (PC1) and the second (PC2) primary components back-projected onto average mean annual precipitation and temperature dimensions of the bioclimatic space between pairs of genera. [file 12870_2021_3151_MOESM4_ESM.pdf]
